# Mechanisms of rainfall-induced landslides and interception dynamic response: a case study of the Ni changgou landslide in Shimian, China

**DOI:** 10.1038/s41598-024-51419-7

**Published:** 2024-01-18

**Authors:** Yu Li, Xuezhi Yang, Xiao Hu, Liyan Wan, Erlong Ma

**Affiliations:** 1 Sichuan Province Engineering Technology Research Center of Geohazard Prevention, Chengdou, 610081 China; 2 Sichuan Geological Environment Survey and Research Center, Chengdou, 610081 China

**Keywords:** Engineering, Civil engineering

## Abstract

Geological hazards, especially landslides and mudslides, are frequent in Caoke County, Sichuan Province, China. In September 2022, the mechanical parameters of the soil were obtained through a basic investigation of the landslide characteristics of Ni changgou. Upon that, the finite element-discrete element method was used to reconstruct the three-dimensional numerical model of the landslide on the right bank of Ni changgou, and the initiation mechanism of rainfall on landslide and the formation of debris flow impact dam process were simulated. Furthermore, the pore pressure, stability coefficient as well as displacement of the landslide body were analyzed. It turned out that with the increase of rainfall intensity, the pore water pressure value also increases, where pore water pressure rises rapidly. the slope is close to the unstable edge, Eventually, it tends to one under rainfall conditions, and due to gravity, the slide of the landslide is induced. The duration of landslide movement is about 200 s, the maximum average velocity of the landslide reaches 4.85 m/s, and the average movement distance is close to 500 m. In addition, this method is applied to the Chutougou debris flow, and the corresponding hazard analysis is added which could better show the treatment and application of debris flow in actual engineering.

## Introduction

Landslides represents a globally prevalent geological hazard, posing a threat to not only agricultural and industrial activities but also the safety of residents. Among various influencing factors, the interaction between rainfall and the initiation of landslides has emerged a pivotal area of research^1–3^. At present, there exists ongoing discourse regarding the mechanisms underlying the relationship between rainfall infiltration and landslide occurrences. Rainfall manifests itself primarily through its intensity and duration. During the initial stages of rainfall, its infiltration of precipitation augments the soil water content in unsaturated areas^4–6^. This, in turn, leads to a reduction in soil shear strength and soil adsorption capacity. In addition, soil deformation alters the seepage process, and prompting key hydraulic characteristics such as soil permeability, porosity and water retention ability to undergo modification in response to changing stress. With the increase of rainfall intensity and time, the instability edge of the landslide draws closer, ultimately inducing the generation of landslide.

Modeling the entire progressive failure caused by rainfall has always been a challenge due to the complex coupling problem of stress and seepage and large soil deformation involved^7,8^. In most cases, the interdependence of infiltration and soil is crucial, as their interaction dictates the hydrodynamic response of unsaturated soil slopes. Simulating the fundamental principles governing landslide initiation under rainfall emerges as an important approach to comprehensively analyze the landslide process. The water-force coupling finite element analysis is an effective method to evaluate landslide, enabling a comprehensively exploration of seepage characteristics and analysis of their influence on landslide deformation^9,10^. However, it can only have a good simulation effect for continuous deformation. Koyama et al^11^. proposed a two-dimensional saturation-unsaturated seepage simulation method based on the finite element method. According to the simulation, they calculated the safety factor of landslide surface under the condition of heavy rainfall. A significant reduction in the safety factor of sliding surface was reported posting rainfall infiltration, ultimately leading to sliding surface and subsequent occurrence of landslide. Ng et al^12^. believed that infiltration or erosion of rainwater leads to soil erosion, resulting in heightened soil pore water pressure or diminished soil matrix suction. This diminishes the shear strength of the failure surface, causing the slope surface to lose equilibrium and precipitating damage and landslides. Extending the exploration of rainfall-induced effects, Yang et al.^13^. studied the slope displacement behavior caused by rainfall infiltration through a series of hydraulic coupling analyses. Their work verified the applicability of slope hydraulic response and deformation, as well as the mechanism of slope deformation and the displacement process of slope over time.

However, practical application demand the integration of the typical two-dimensional rainfall-induced landslide with the complicated three-dimensional geology. Within the realm of rainfall modeling, the Van Genuchten (VG)^14^ model is one of the initial and successful methods to characterize the transient infiltration process of water in unsaturated soils. The widespread utilization of the VG model in practical applications is attributed to the clear physical meaning and measurability of its variables. In this paper, the vg model is employed to calculate the seepage field under the action of rainfall, followed by the application of an equivalent simplified force to the continuous–discontinuous model to calculate the seepage—stress field.

Currently, the rainfall models proposed by scholars predominantly rest on continuum mechanics, which falls short in analyzing the damage characterized by large deformation and the process of landslide movement triggered by rainfall^15–17^. Discrete element method has gained significant traction in studying the movement processes of slope landslide and debris flow^18–20^. Most existing landslide and debris flow models are based on the mathematical framework proposed by Zienkiewicz^21^, with further refinements based on the framework made by Biot^22^. Several fundamental models, such as discrete element method (DEM) ^23,24^smooth particle Fluid dynamics (SPH)^25–27^ method and material point method (MPM)^28^, are applied to landslide and debris flow models. Among these models, DEM is particularly well-suited for simulating dry particle flow, while MPM is mainly used for solving continuous solid mechanics problems^29^. In contrast, SPH is a grid-free method that can be easily used for large deformations. The SPH model has been widely used to analyze the dynamic processes associated with plastic or viscoplastic flow, and has shown good potential in modeling flow-structure interaction problems^30,31^. Therefore, this paper adopts the sph model to simulate the motion pattern of debris flow.

The debris flow initiated by rainfall-induced landslide exhibit large fluidity and can traverse long distance, causing damages to nearby infrastructure and posing a substantial threat to local residents^32,33^. The construction of retaining DAMS can reduce the harm of debris flow. However, the complexity of the debris flow impact process presents challenges in accurately determining the maximum impact force of debris flow on the structure. Bi et al.^34^ used DEM to calculate the impact force on the baffles under different configurations, providing insights into the influence of the number of baffle rows and column spacing on the impact force. Moreover, the lattice Boltzmann method (LBM)^35^ and material point method (MPM)^36^ were used to numerically analyze the deceleration effect of baffle on the particle flow and the influence of particle flow on the baffle. In addition, SPH, as a continuum method, proves efficient in calculating large-volume particle flows. Therefore, the SPH method has been widely used to simulate the dynamic behavior of particle flows in irregular terrain, encompassing scenarios such as debris flow and landslide. Zhang and Xiao^37^. simulated the propagation and entrainment of massive debris flow using SPH method. These results not only help to further understand the water flow dynamics, but also provide a scientific basis for disaster assessment. Ren and Shu^38^ proposed a new simulation algorithm designed to address fluid–structure coupling problems, particularly within the context of natural phenomena such as mudslides. In this approach, the fluid part (debris flow fluid) was simulated under the framework of smooth particle fluid dynamics (SPH) method, while the solid part (downstream obstruction) was simulated using the finite element method (FEM). The SPH-FEM coupling method offers a robust solution for the simulation of fluid-structure coupling problems. This paper leverages the SPH-FEM coupling method to study the related parameters associated with debris flow and dam. The finding of this study can provide a reliable basis for the engineering design of baffle array.

In this study, a continuous–discontinuous coupling method is employed to analyze variations in slope water pressure and displacement of the slope under different rainfall conditions through numerical simulation. This approach unveils the intricate mechanism governing landslide sliding. The simulation landslide sliding is achieved by converting the seepage force into discrete element nodes. Subsequently, the entire process of landslide sliding under the influence of rainfall, culminating the impact with the dam, is simulated. A risk assessment is conducted to gauge the hazard intensity of debris flow, considering factors such as mud depth. This assessment provides a foundation for disaster analysis and aids in the design of effective measures for disaster reduction.

## Study areas

### Overview of the landslide area

**Figure 1:**
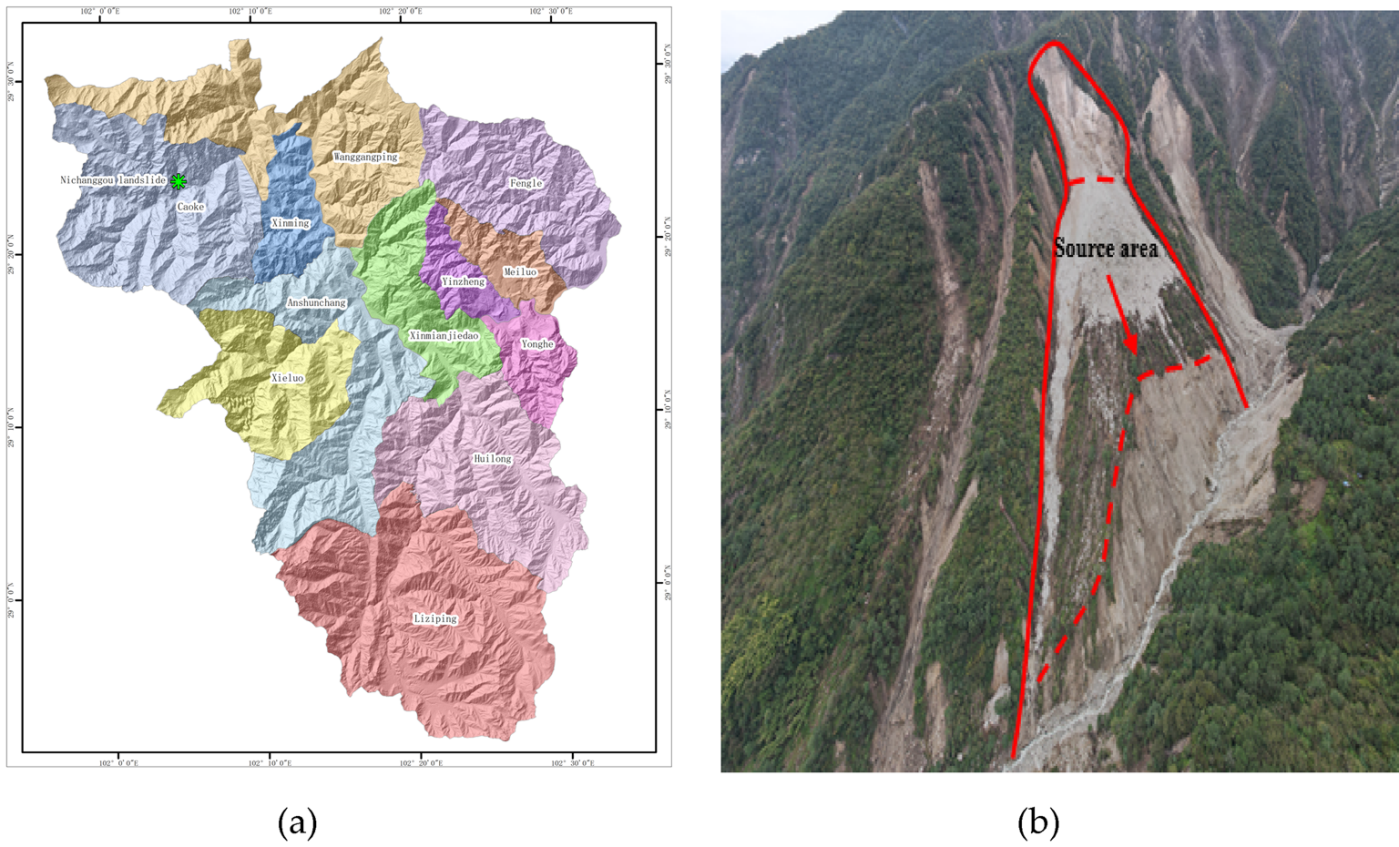
(**a**) Location map of the study area. Primary topographic landslide parameters such such as relative relief was derived using ArcGIS(10.6) software in a GIS environment using 12.5 DEM data. 12.5 DEM data Sources ASF Data Search (alaska.edu). (**b**) Remote-sensing image of Ni changgou landslide.

**Figure 2:**
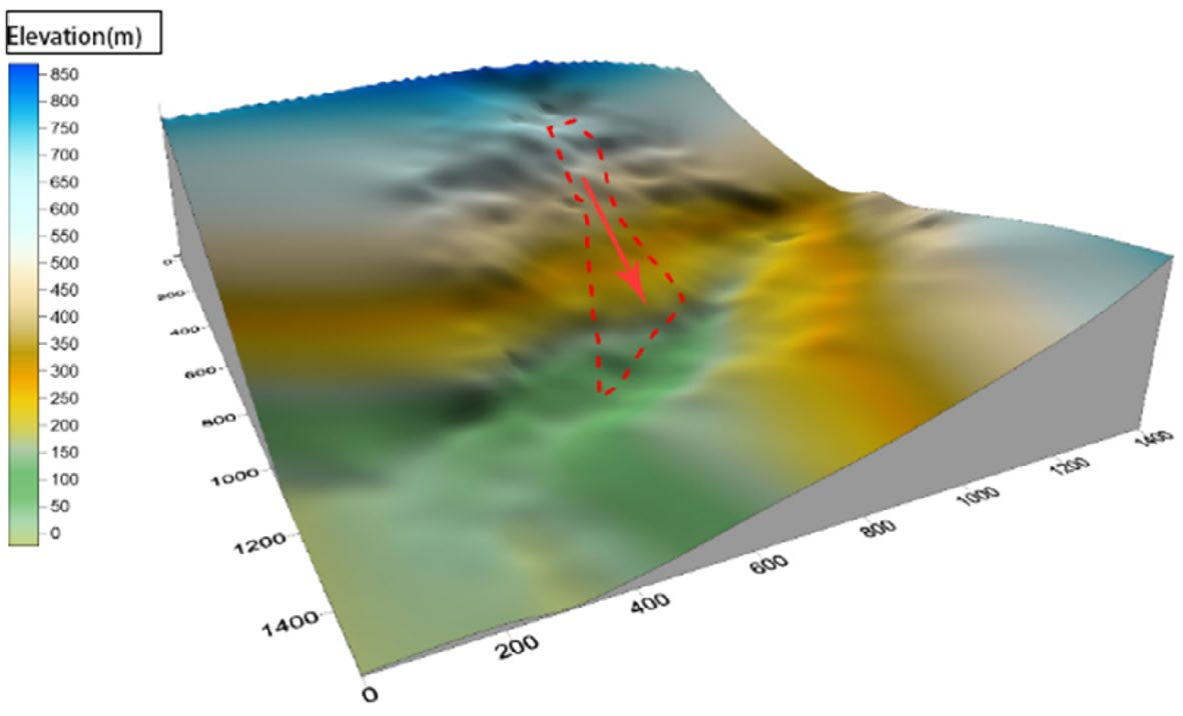
Three-dimensional topography of the landslide.

Ni changgou landslide, a first-class tributary of the left bank of Tian Wan river, is located in group 2 of He Ping village, Cao Ke township, Shimian county (Fig. 1a), which is a first-class tributary of the left bank of Tian Wan river, about 5 km from Cao Ke township, Asbestos county in the north, about 59 km from Asbestos Shimian county. The geographic coordinates of the landslide center are given as: 102°5′44.79″ E, 29°23′33.99″ N. There is a village road passing in the middle reaches of Ni changgou, and the traffic conditions are general. And the lower part is a landslide area with a steep topographic slope of 60°–70° (Fig. 1b). As shown in Fig. 2, This shows that the Ni changgou landslide with an elevation between 0 and 850 m.

### Geomorphology

**Figure 3:**
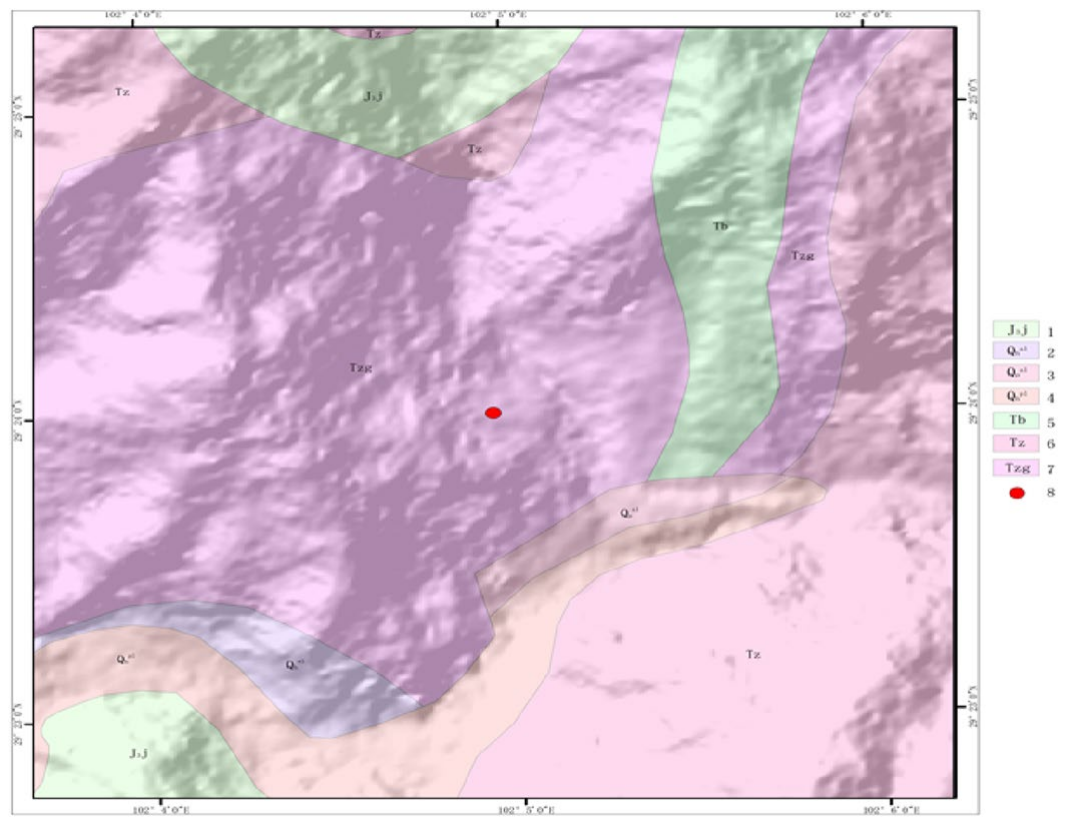
The geological map of the study area: (1) Late Jurassic intrusive metamorphic rock; (2)–(4) fourth series loose accumulation; (5) metamorphic rocks of the Upper Triassic Spinzigou Formation; (6) metamorphic rocks of the Upper Triassic miscellaneous valley brain formation; (7) metamorphic rocks of the Upper Triassic Zagashan Formation; (8) Ni changgou landslide.

The stratigraphic lithology of the study area range is shown in Figure 3, which is widely distributed with metamorphic rocks (Tzg) black cloud schist of the Upper Triassic Zagashan Formation. The lithology is greyish-white, made up of massive marble and occasionally interspersed with quartzite and quartz schist, while piling up a large amount of loose material, and if heavy rains occur frequently, landslides will appear along the channel to form mudslides.

### Landslide characteristic analysis

**Figure 4:**
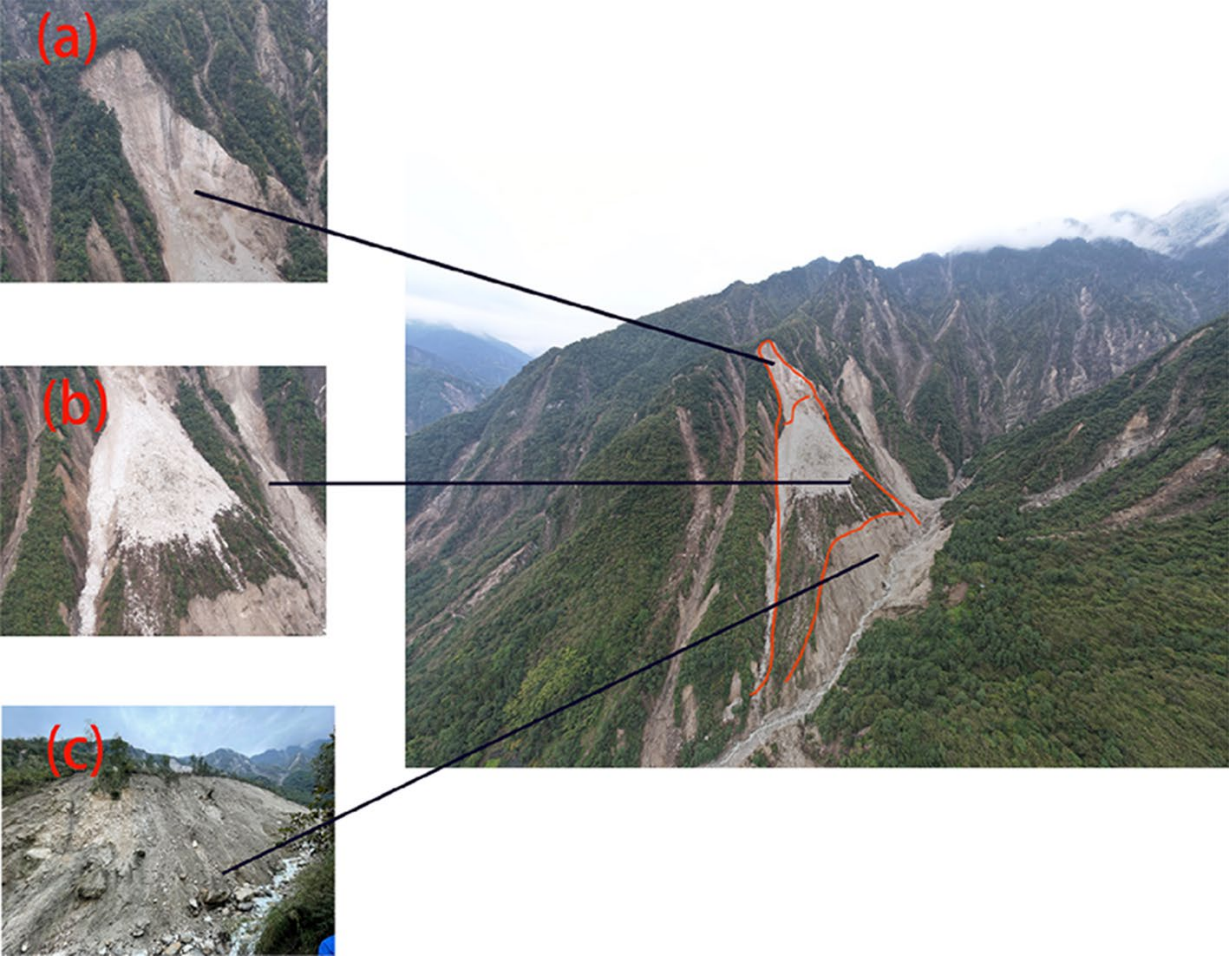
The characteristics of Ni changgou landslide are detailed as follows: (**a**)the slide source area. (**b**) The accumulation zone. (c) The collapse zone.

**Figure 5:**
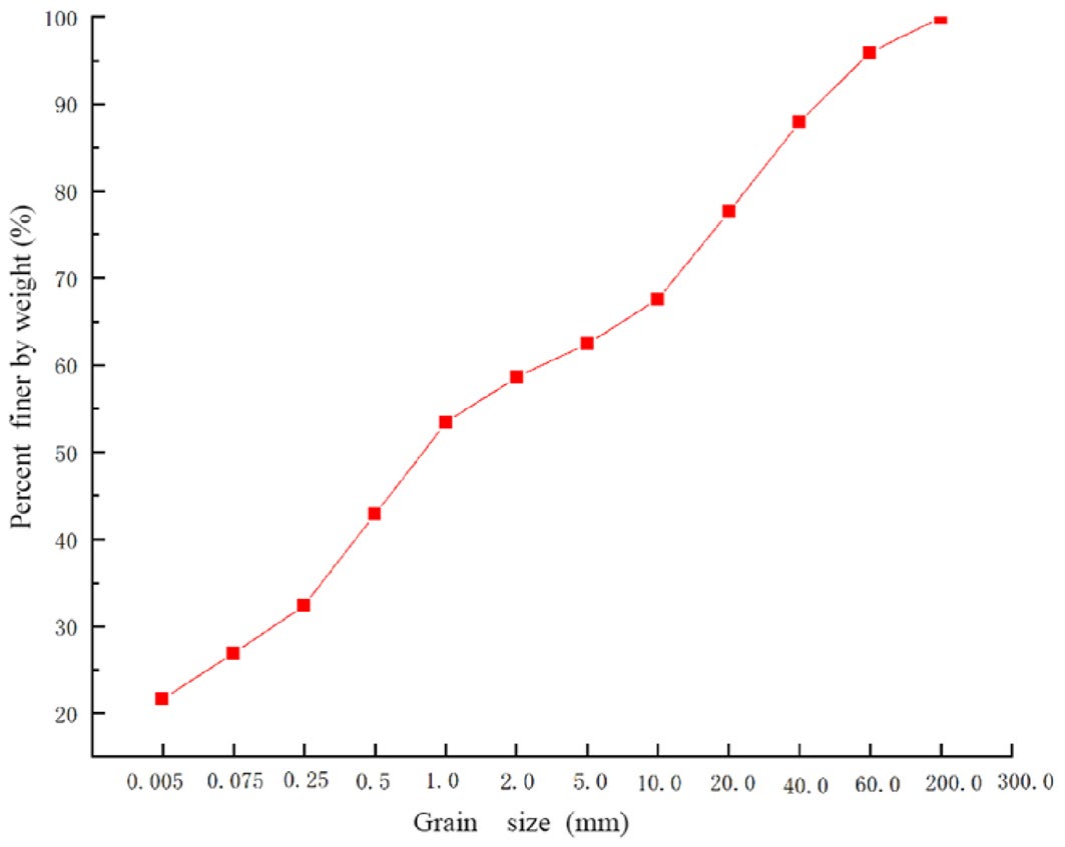
Grain size distribution of the silty clay.

This landslide is not a newly-formed area, as a small-scale landslide occurred before the rainfall, and heavy rain triggered a large-scale avalanche slide in the upper part of the landslide. The cause of the landslide is the loose soil layer and barite on the upper part of the slope, along the contact surface between the surface layer of strong weathering zone and the intact bedrock landslide, the shear outlet is located in the middle of the slope. The landslide body is 1060 m long, 180 m wide on average, 1–8 m thick, with a volume of 576,000 m^3^. According to the deformation characteristics of the landslide, it is divided into three sub-areas: slip source area, accumulation area and collapse area (Fig. 4). Figure 5 shows the photo of the silty clay in the landslide site.

**Figure 6:**
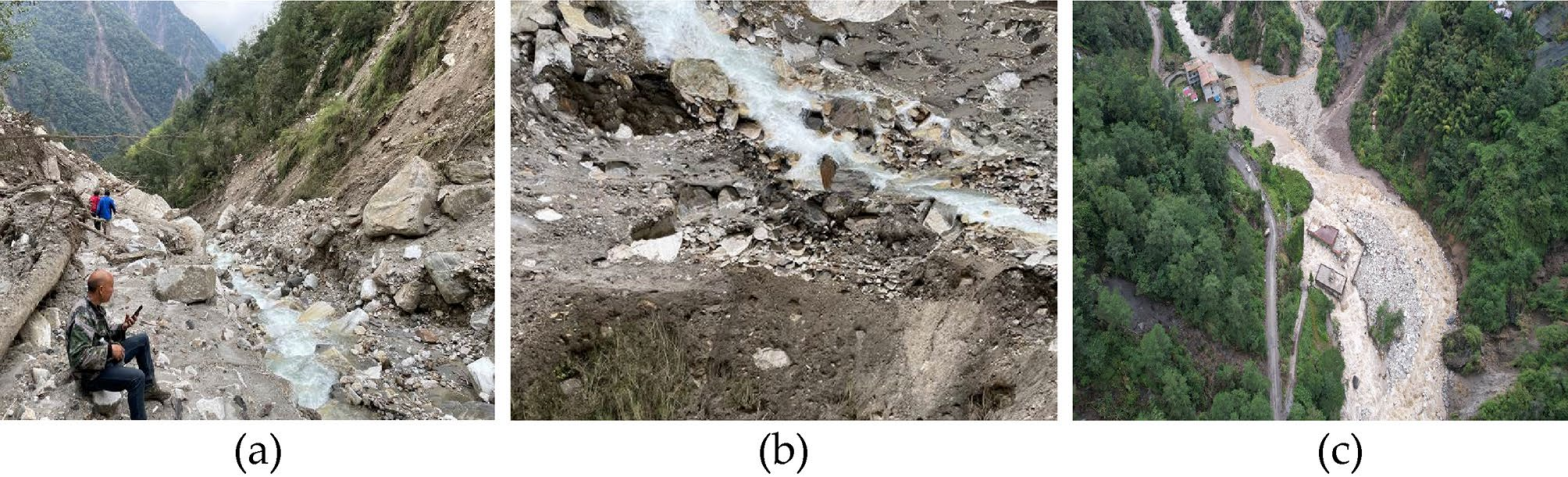
Damages caused by the Ni changgou landslide: (**a**) uncovering bottom scouring of trench; (**b**) small amount of trench accumulation; (**c**)threatened objects by Ni changgou landslide.

As a result of heavy rainfall, the landslide slid along the trench, and under the influence of heavy rainfall and surface water flow, the landslide flowed into the downstream trench (Fig. 6a), The area from the back edge of the avalanche slide accumulation to the top of the slope makes up the slip source, with an elevation range of 2175–2370 m and relative height difference of 205 m. A steeper topography slope of 60° is observed with downhill length of about 184 m, the average width is about 120 m, and the area is 2.4 × 104 m^2^. The slope surface leaves a small amount of landslide accumulation, with a thickness of 0.5–1 m, which also had a small amount of accumulation (Fig. 6b) The accumulation area is located in the middle of the slope, the elevation range is from 1850 to 2175 m, the relative height difference is 325 m, the terrain slope is steep with a 45° average slope. The area of the accumulation area is 15.5 × 104 m^2^ with a length about of 740 m and an average width of about 210 m, the volume of the accumulation body is about 46.5 × 104 m^3^ as its thickness is 2–5 m. Most of the accumulation body stays on the slope surface while some of them enters the trench, the accumulated materials are mainly powder clay with crushed stones, of which the content of crushed stones is about 40%.The size of the general particle is 5–60 cm, the main composition is mainly marble without sorting and rounding. And its structure is loose, which eventually causes serious damage to the surrounding farmland, vegetation, houses and infrastructure (Fig. 6c). The collapse area is located on the front edge of the slope, where the terrain slope, reaches a steep degree of 70°, as a result of rainfall scouring effect. The downhill length of the collapse area is about 140 m long, the average width is about 440 m, and the area is 6.2 × 10^4^ m^2^. The material composition is mainly powder clay sandwiched by crushed stone, with a thickness of 1–3 m and a volume of 11.1 × 10^4^ m^3^. Due to mudflow and flood scouring the slope foot the collapse area was seriously damaged, piled up in the trench.

### Rainfall characteristics

**Figure 7:**
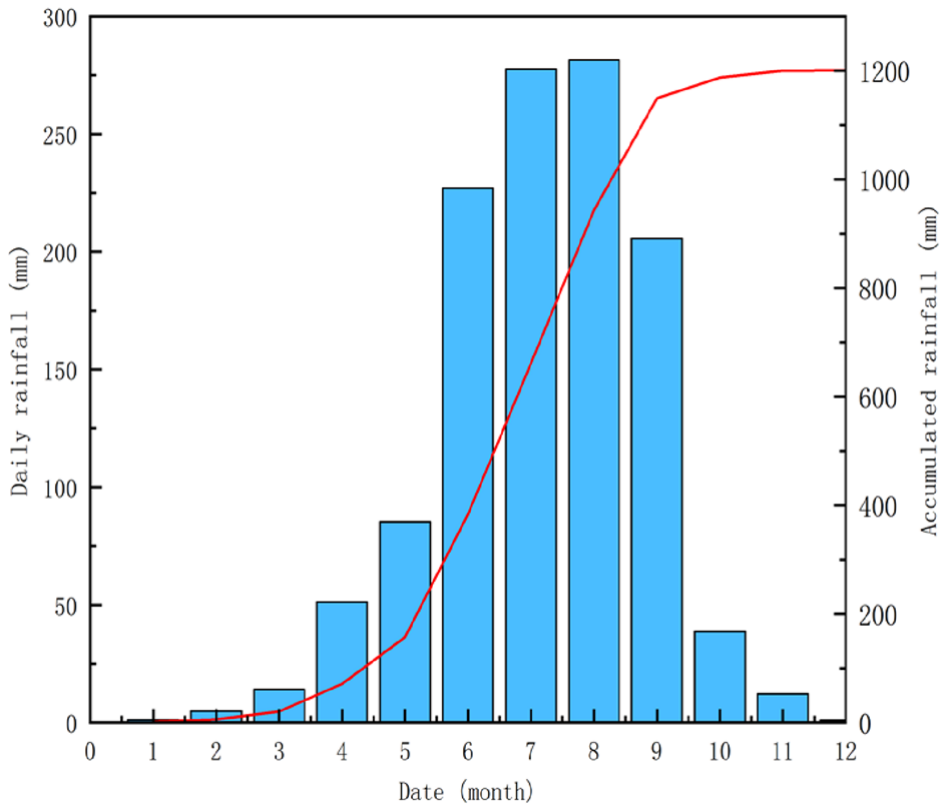
Accumulated and daily rainfall data of Ni changgou landslide.

The climate of the study area is mountainous with a subtropical monsoon climate as its basal zone, where there is an alpine boreal climate in the plateau above 3500 m above sea level, and a subtropical monsoon climate below 3500 m. The average annual precipitation for several years is 1200.9 mm (displayed in Fig. 7), but it is unevenly distributed in space and time as the precipitation is mainly concentrated from May to September, accounting for 86.4% of the annual precipitation; meanwhile, there is more rainfall in the mountains than in the river valleys, and it mostly occurs as heavy rain or showers.

## Methodology

### Study on the mechanism of landslide initiation by rainfall

Rainfall infiltration is a continuous dynamic process, which performs a significant impact on landslide initiation, while the intensity of rainfall affects the speed of landslide initiation. Rainfall_triggered landslide initiation can be divided into two stages. In the first stage, the infiltration intensity of the soil is higher than the rainfall intensity, thus the rainfall will not form seepage, as it belongs to a completely unpressurized state. In the second stage, the infiltration intensity of the soil is less than the rainfall intensity, the soil absorption rate is higher, rainwater will produce runoff on the surface, then the pressurized state occurs. The greater the rainfall intensity, the smaller the rainfall infiltration rate, the longer the duration of rainfall affecting induced landslides, and the greater the water content leading to soil destabilization.

Generally, the discrete element method is widely used in landslide initiation related research works, compared with traditional mechanics, discrete elements have the advantage of simulating large deformation, adopting discrete elements, considering the effect of rainfall, modeling with equivalent depth of infiltration ^39^, and applying flow-solid coupling ^40^. In the process of discrete element simulation of landslides, the calculation of stress resulting from the excessive amounts of elements often causes large deviations. Therefore, the method of continuous–discontinuous coupling calculation can be used to simulate the landslide initiation, in which the soil is treated as a continuous medium while the damaged soil is regarded as discrete elements. The infiltration of rainwater is quite difficult for the discrete element simulation, however, it is much easier in the continuous medium simulation. If the two are combined, it can simulate both the landslide initiation caused by rainfall and the damage effect of continuous medium under rainfall in an effective way. The results of this study are believed to provide a valuable reference for the application of continuous–discontinuous numerical simulation in geotechnical engineering.

### Rainfall model

In this contribution, we propose a continuous-discontinuous coupling model with the relative processes of different rainfall amounts, of which the saturated and unsaturated pairs of infiltration flows are considered in order to continue the simulation of the landslide initiation process under the influence of rainfall after the completion of rainfall infiltration, and to save the results of rainfall calculations into another storage node that opened within the cell.

The rainfall loads are transformed into equivalent forces applied to the continuous model and the discontinuous model. The simulation of the model is divided into rainfall process and landslide initiation process. Since rainfall is a long period process, deformation is usually not considered in the analysis, the theory of saturated–unsaturated seepage is employed instead to record the pore pressure field and safety factor. The landslide initiation process allows the model to deform freely, during this process, the areas where damage may occur get discretized, it gives possibility to perform a continuous–discontinuous coupling computational analysis for rainfall landslides.

The rainfall process can be simulated according to saturated–unsaturated theory, of which the unsaturated permeability coefficient is generally used to reflect the change process with soil-water characteristic curve. According to the relationship between soil water content and permeability coefficient, the Van Genuchten (VG) model ^20^ between them can be represented as follows:

1 $$\uptheta ={\theta }_{r}+\frac{{\theta }_{s}-{\theta }_{r}}{[1+{\frac{p}{{\text{a}}})}^{{n}^{\mathrm{^{\prime}}}}{]}^{{m}^{\mathrm{^{\prime}}}}}$$

where θ denotes the volumetric water content of the soil; ${\theta }_{r}$ refers to the residual volumetric water content of the soil; ${\theta }_{s}$ is the saturated volumetric water content of the soil; $p$ represents the pore water pressure (Pa) of the soil; ${\text{a}}{,m}^{\mathrm{^{\prime}}},{n}^{\mathrm{^{\prime}}}$ are the fitting parameters, and generally, ${\text{a}}$ takes the value of 100, ${m}^{\mathrm{^{\prime}}}$ takes the value of 1, and ${n}^{\mathrm{^{\prime}}}$ takes the value of 2.

Considering the relationship between volumetric water content and saturation in the soil, $\uptheta ={\text{ns}}$, where ${\text{n}}$ is the porosity and ${\text{s}}$ refers to the saturation. The relationship between negative pore water pressure and saturation can be formulated by bringing in the above equation.

2 $${\text{s}}={s}_{r}+\frac{1-{s}_{r}}{[1+{\frac{p}{{\text{a}}})}^{{n}^{\mathrm{^{\prime}}}}{]}^{{m}^{\mathrm{^{\prime}}}}}$$

where ${\text{s}}$ means saturation; ${s}_{r}$ denotes residual saturation, and the rest of parameters mean the same as above. The relationship between pore water pressure permeability coefficient ${k}_{w}$ and soil saturation permeability coefficient ${k}_{s}$ an be specified as follows:

3 $${k}_{w}=\frac{{k}_{s}}{1+{\text{a}}[{\frac{({u}_{a}-{u}_{w}}{{p}_{w}{\text{g}}})]}^{n}}$$

where ${u}_{a}$ denotes the intra-pore gas pressure, ${u}_{w}$ denotes the pore water pressure, ${u}_{a}-{u}_{w}$ negative pore water pressure absolute value, ${p}_{w}$ denotes the fluid density, g denotes the acceleration of gravity, a, n is the fitting parameter, for general soil body a take the value of 0.1, n take the value of 2.

From the above formula, it can be seen that as the absolute value of pore pressure gradually decreases, that is, the water content gradually increases, the permeability coefficient gradually increases, thus it is considered that the permeability coefficient reaches its maximum value when the soil is saturated and remains unchanged.

### Continuous–discontinuous equivalence method

An equivalent simplification is needed with the saturated-unsaturated infiltration calculations, it aims to apply the rainfall effects to the particles of discontinuous mechanics. First of all, the increase of the gravitational acceleration in the soil, that results from rainfall infiltration, must be considered. The soil gravity ρ affected by rainfall can be calculated through the following equation.

4 $${\uprho ={\uprho }_{d}+\uptheta \cdot \uprho }_{w}$$

The equation ${\uprho }_{d}$ represents the dry weight of soil; $\uptheta$ refers to the volumetric water content; ${\uprho }_{w}$ denotes the weight of water. In saturated soil, the direction of seepage force keeps consistent with the direction of seepage as the presence of head pressure difference can produce seepage force on the soil, while the effect of seepage force in unsaturated soils is neglected. The equivalent infiltration force ${{\text{F}}}_{f}$ applied in the saturated region is:

5 $${{\text{F}}}_{f}=J\cdot V={\upgamma }_{w}\cdot i\cdot V$$

The variable $J$ is the infiltration force; $V$ denotes the volume of the unit grid or particle cell; ${\upgamma }_{w}$ represents the heaviness of water (N/m^3^ ), $i$ refers to the hydraulic gradient, and its magnitude value is the ratio of the difference between the water potential at two points in the soil and its infiltration distance. The magnitude of the buoyancy force on the particle under the action of saturation can be calculated by the following equation:

6 $${{\text{F}}}_{f}=pgV$$

In the saturated state of the soil, there is a certain degree of discounting in its physical and mechanical parameters due to the softening effect. In the discontinuous model, the effects of rainfall loading on the increase in soil weight, seepage and buoyancy forces, and parameter discounting are considered, and the equations are Eqs. (4)–(6). In the continuous model, since it is not the main area where debris flow initiation occurs, only the effects of soil weight increase and parameter discounting are considered to ensure the reasonableness of the calculation efficiency.

### SPH-FEM theoretical model (blocking dynamic response model)

Landslides are initiated along gullies to form mudflows, which are usually composed of large amounts of sediment and rock and are excited to be born under extreme climatic conditions such as heavy rainfall in valleys. Studies on modern debris flow have evolved from traditional single-discipline and single-method studies to interdisciplinary combination and multi-method research. Several representative studies are specified as follows. A new coupled SPH-DEM method by Hara ^41^ was proposed to model the coupled motion problem of fluids and solid particles, which considered the capillary and viscous interactions between the fluid and particles. Similarly, Xu^42^ used SPH-DEM coupling to simulate the 1963 Vajont reservoir landslide surge in Italy, and it turned out that the final accumulation pattern of the calculated landslide is more consistent with the actual accumulation topography. Also, a SPH-DEM coupling method by Lin tal^43^ was employed to simulate the occurrence and propagation of discrete soil landslide surge waves, with which the landslide scale and surge velocity correlation curve were developed. The coupling methods consist of smooth particle hydrodynamic method (SPH), finite element method (FEM), of which the SPH represents the fluid, while the FEM unit refers to the topography and structure. For finite and discrete elements, the SPH-FEM coupling model is a better choice to simulate the contact process between debris flow and rigid structural units.

In this contribution, an experience based SPH-FEM model is used to simulate the stopping response of the debris flow to the dam. The fluid in the debris flow is calculated using SPH, and the debris flow trench as well as barrier structure are carried out using FEM, upon which a complex dynamic interaction model of particle-fluid-structure and a simplified trench dam model can be established to illustrate the process of debris flow impact with the dam.

### Model establishment

**Figure 8:**
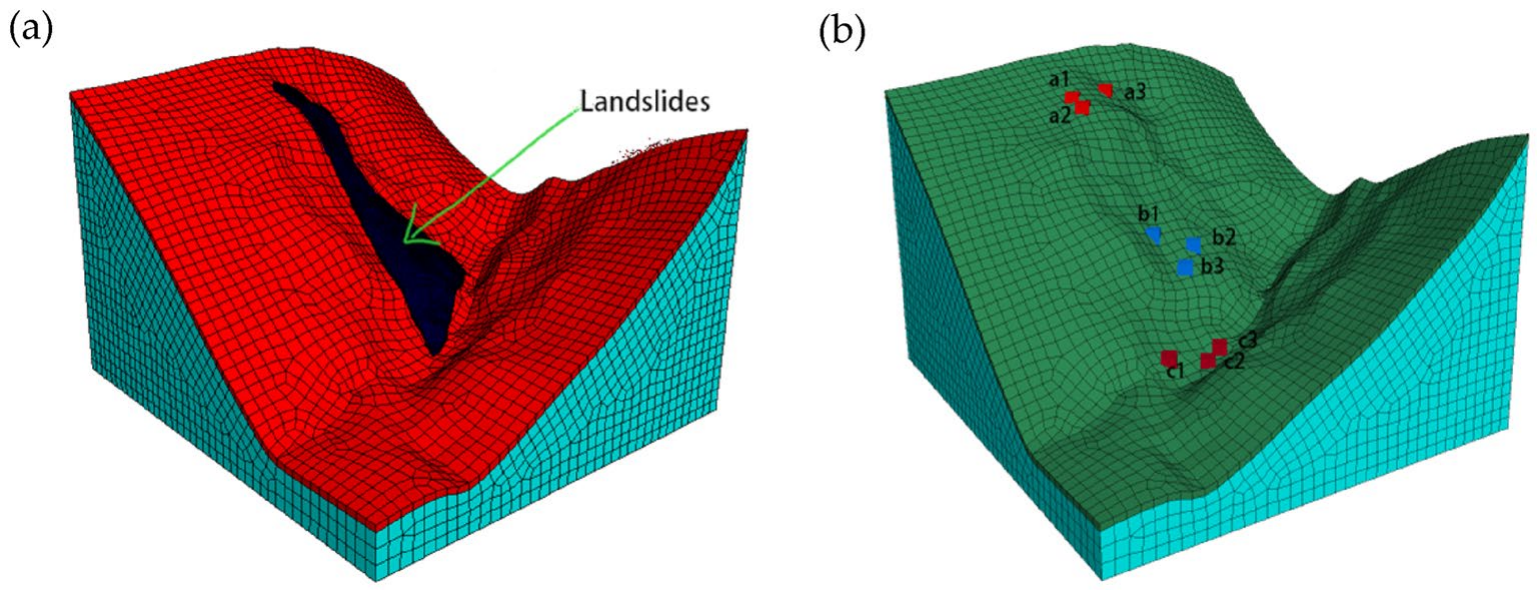
Initial model building: (**a**) model meshing; (**b**) arrangement of slope monitoring points.

The calculation model is based on the Ni changgou landslide model, and the slope calculation model is divided by hexahedral mesh, with 9009 nodes and 43,888 cells in total (Fig. 8a). In order to analyze the infiltration damage in the upper, middle, and lower parts of the landslide, 9 points are now implanted and the location of the monitoring points (Fig. 8b). Ni changgou landslide with the micro-mechanical parameters shown in Table 1.

**Table 1:** Landslide-related parameters.

Parameters	Landslide	Bedrock
Modulus of elasticity (MPa)	3.0	3.8
Poisson’s ratio	0.3	0.35
Density (kg/m^3^)	2105	2650
Cohesion (kPa)	10.3	10.5
Friction angle (°)	26.5	28
Penetration rate	1.81 × 10^–8^	1.0 × 10^–8^
Unit time step (s)	3.0 × 10^–4^	3.0 × 10^–4^
Time step (n)	5.0 × 10^5^	5.0 × 10^5^

**Figure 9:**
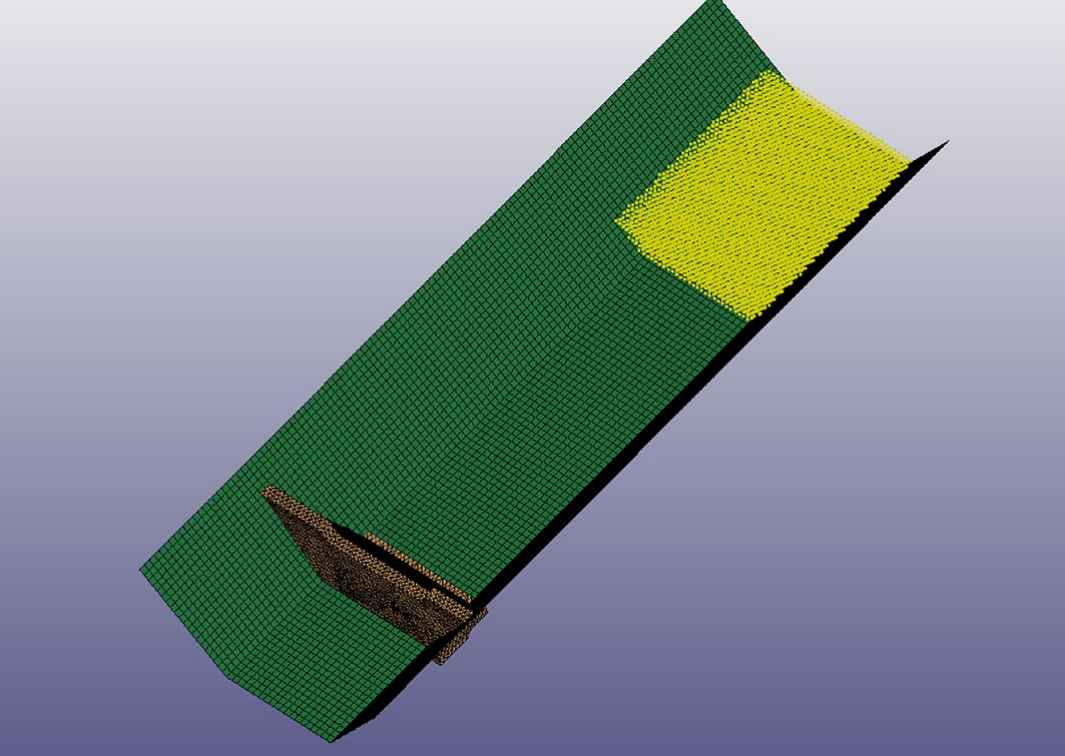
Simplified model of gully dam debris flow.

Landslides can easily cause mudflows along trenches, and the impact of mudflows on control engineering structures is indeed an important consideration. At present, the impact effect of mudflow on the dam body belongs to a complex process due to the complex topography and large grid calculation. In most research literature, it is simplified by selecting the SPH-FEM method and using rigid body cells to facilitate the simulation on the bottom of the trench as well as the rock bodies on both sides (Fig. 9). Considering the mudflow material source of this area is mainly rainfall, the mudflow fluid is simulated by SPH particles, with a total of 9219 particles. The gravitational acceleration used in the calculation is 9.8 m/s^2^. For the debris flow channel, the simulation is performed with rigid shell material, which can significantly reduce the time of simulation, and the shell cells are used this turn, with a total of 5000 shell cells. The relevant parameters are shown in Table 2.

**Table 2:** Simulation of parameters related to heavy rainfall.

Rainfall program	Rainfall time (days)	24 h rainfall (mm)
Rainfall Program I	14	180
Rainfall Program II	14	260

### Calculated solutions

**Figure 10:**
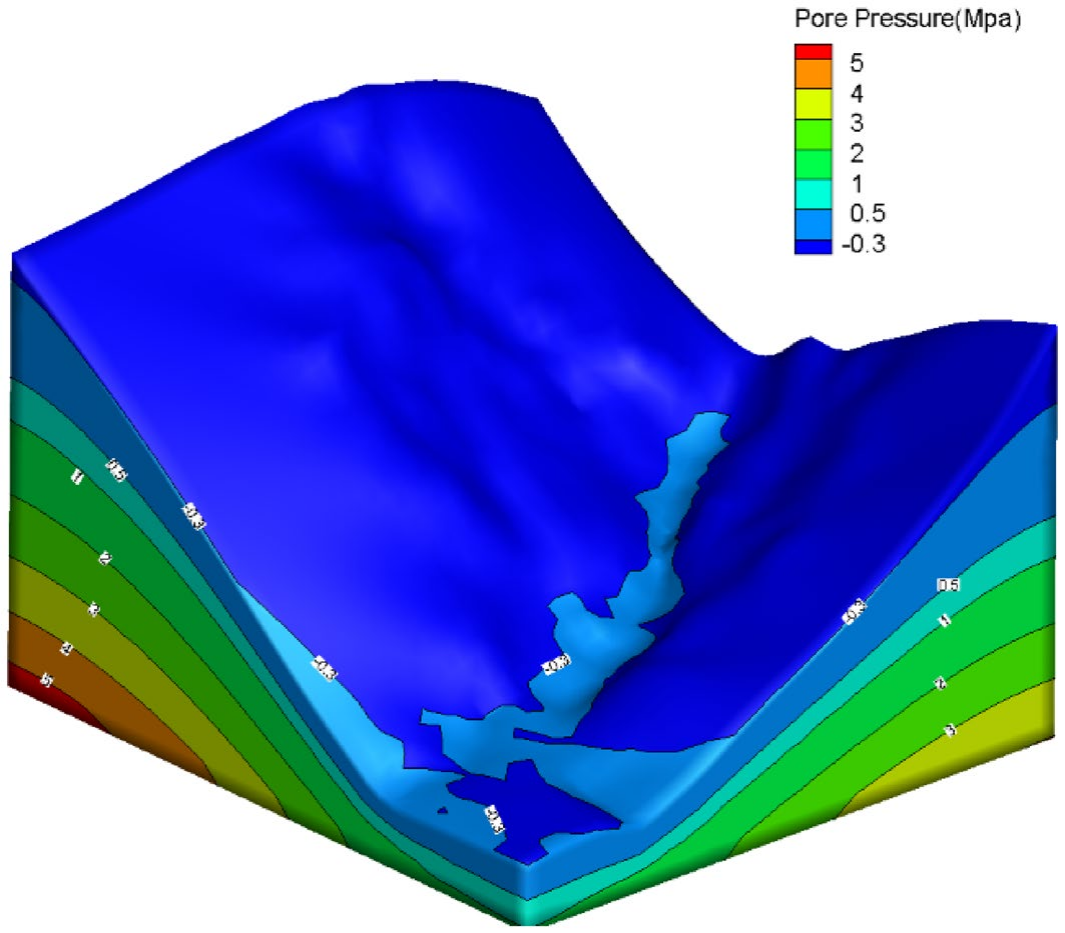
Initial calculation of model pore pressure distribution.

To induce landslide initiation mechanism with rainfall, this contribution simulates rainfall landslides by establishing continuous–discontinuous coupling. After the calculation of initial seepage, the calculation results are used in the coupling model, and a changing process of seepage field is derived from different rainfall amounts, and continuous–discontinuous coupling landslide analysis under rainfall conditions is carried out on the targeted landslide. The initial pore pressure field is set at the boundary of both sides of the model (Fig. 10), and then the saturation–disaturation calculation is used to stabilize the generation. The pore pressure and safety coefficient changes are determined by the coefficient parameters that given by different rainfall amounts. Rainfall is classified according to the meteorological classification standard in China: 24 h rainfall <10 mm is considered as light rain; 24 h rainfall between 10 and 24.9 mm is considered as medium rain; 24 h rainfall between 25 and 49.9 mm is considered as heavy rain; 24 h rainfall between 50 and 99.9 mm is considered as heavy rain; 24 h rainfall between 100 and 249.9 mm is considered as heavy rain; 24 h rainfall ≥ 250 mm is defined as very heavy rainfall, considering the different conditions of rainfall, the selection is heavy rainfall and very heavy rainfall is shown as Table 2.

## Results

### Effect of rainfall intensity on seepage field

**Figure 11:**
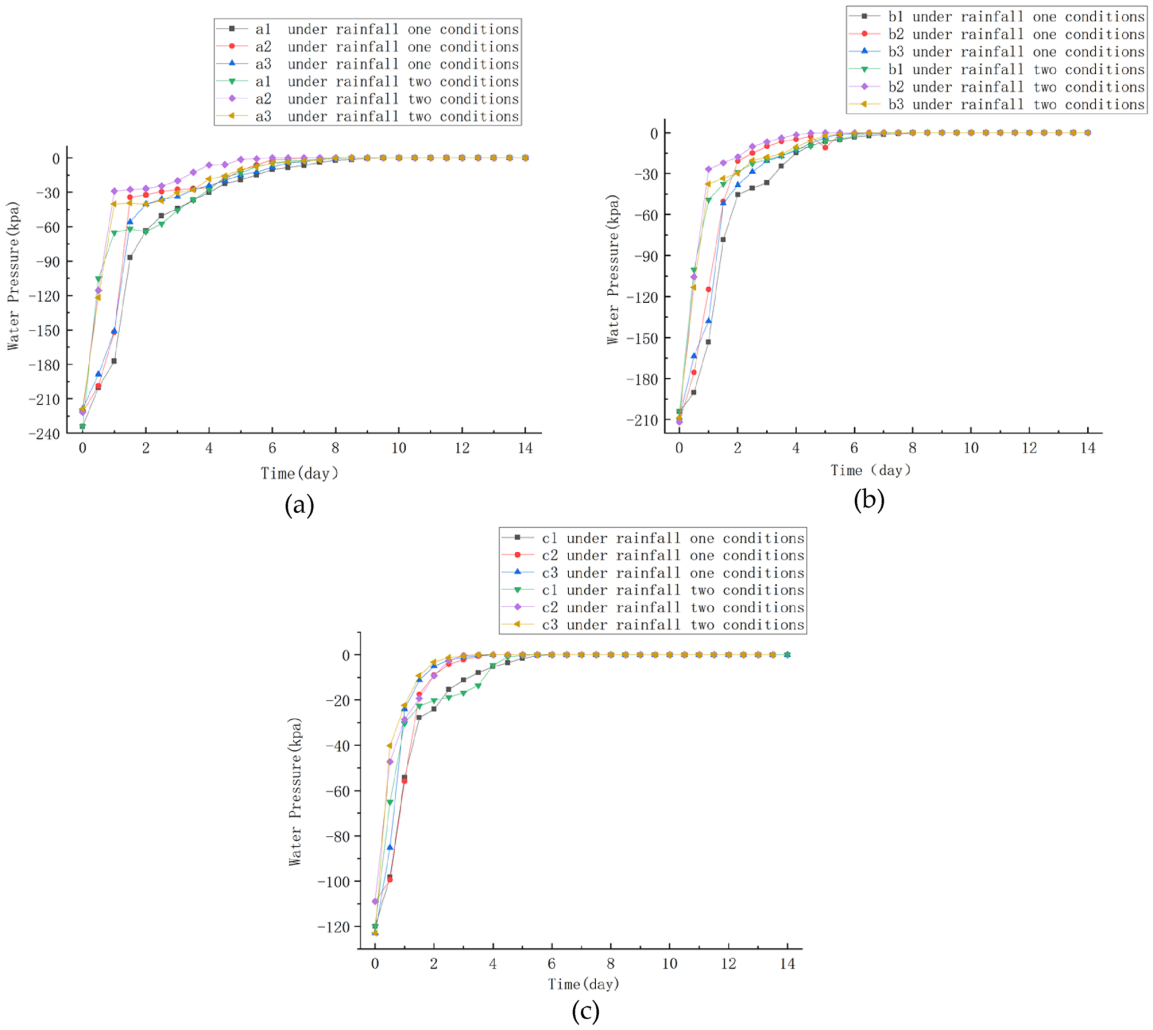
Monitoring point water pressure change: (**a**) A1, A2, A3 at the top of the slope; (**b**) b1, b2 and b3 in the middle part of the slope; (**c**) The foot of the slope is at the c1, c2, c3 nodes.

**Figure 12:**
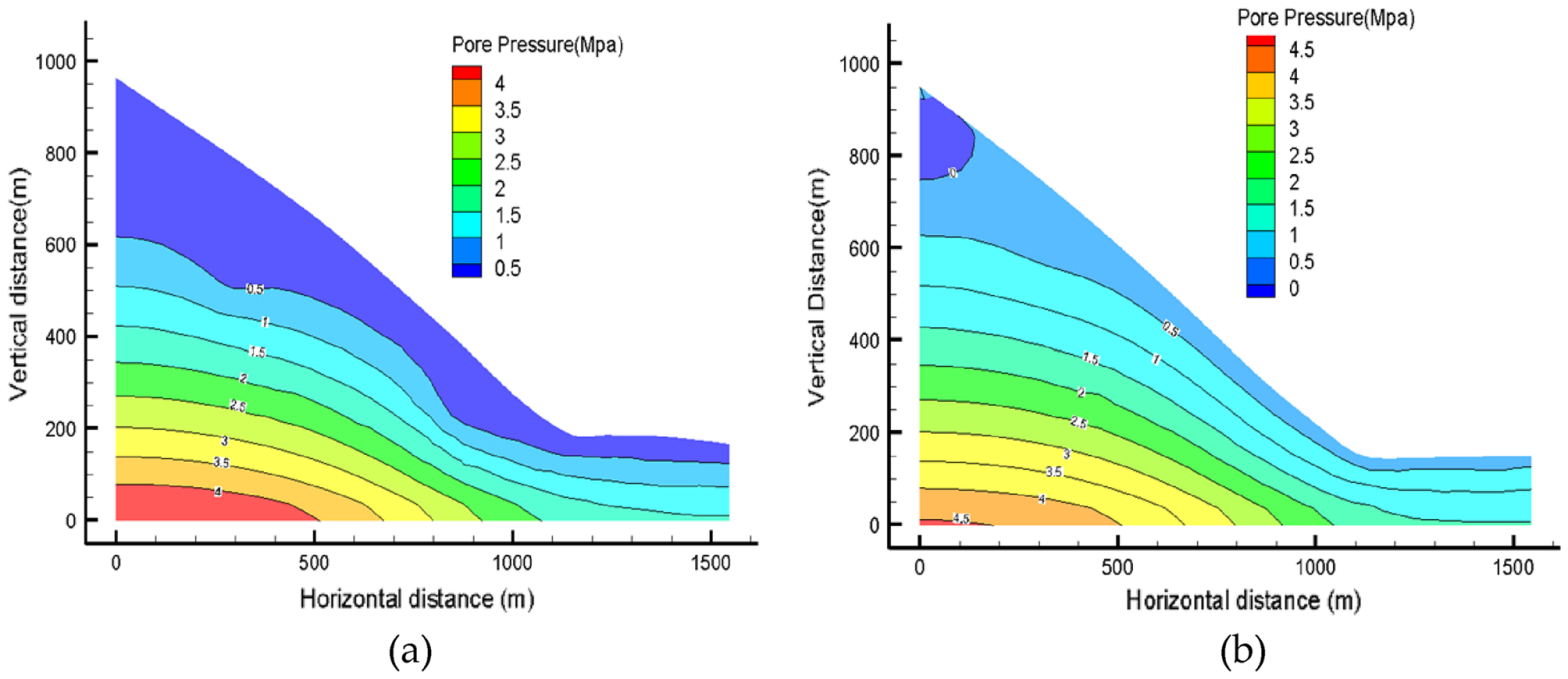
Pore water pressure of rainfall: (**a**) pore water pressure of the first type of rainfall; (**b**) pore water pressure of the second type of rainfall.

Under the two rainfall intensity conditions of scenario I and scenario II, the pore water pressure changes within the distribution were recorded by setting different monitoring point maps on the slope. It can be clearly observed that as the rainfall intensity increases, the pore water pressure values at the preset monitoring points also increase. In the unsaturated soil, the soil gradually approaches the near-saturated state and even reaches the saturated state. Among them, the pore water pressure values at nodes a1, a2, and a3 at the top of the slope changed most dramatically (Fig. 11a), followed by nodes b1, b2, and b3 in the middle of the slope (see Fig. 11b), while nodes c1, c2, and c3 at the foot of the slope (displayed in Fig. 11c), changed most slowly. In addition, the rate of change of pore water pressure values at the monitored nodes was also significantly faster under the condition of rainfall scenario 2 with greater rainfall intensity. The seepage field clouds calculated in the two rainfall scenarios show that under the rainfall duration of 14d (Fig. 12a, b), the saturated zone performs a diffusion from the slope surface to the interior of the slope, and the area of the slope affected by rainfall gradually expands with the rainfall, and the positive pore water pressure first appears at the location of the slope surface, and the groundwater table surface at the foot of the slope has an obvious rise. In the rainfall scenario II with greater rainfall intensity, the saturated area is significantly larger than that in scenario I.

### Analysis of edge stability under rainfall conditions

**Figure 13:**
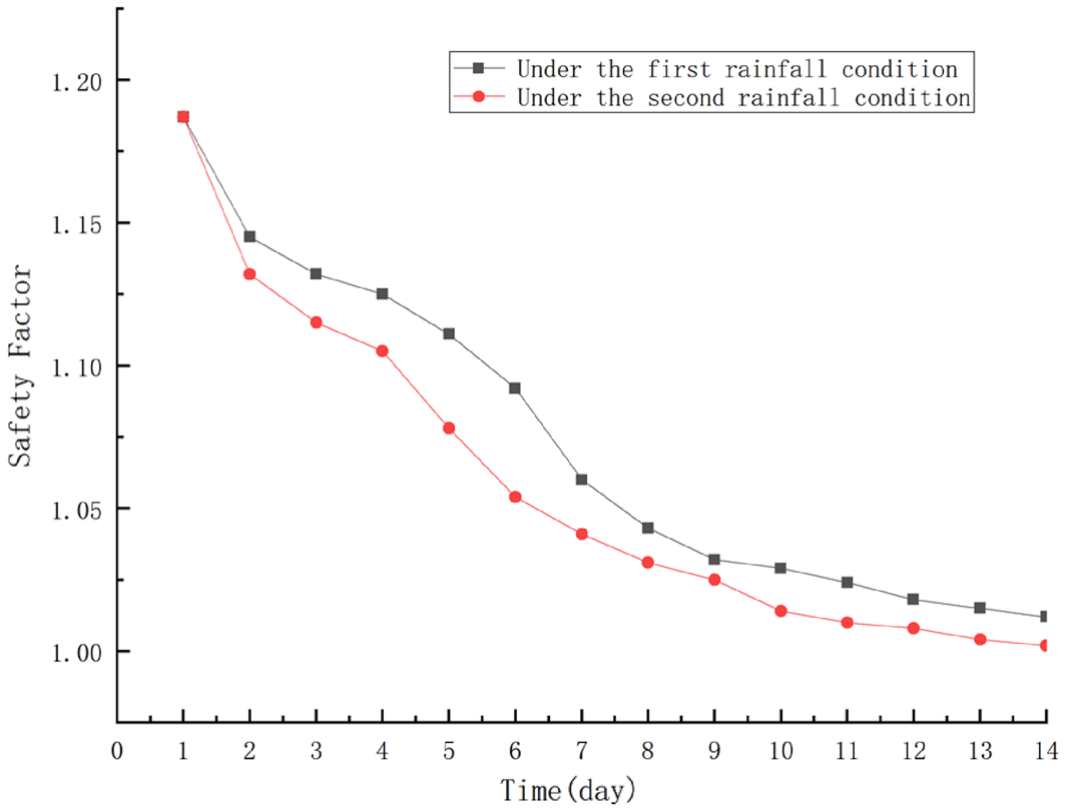
Variation process of side slope coefficient under rainfall condition I and rainfall condition II.

The transient analysis method was adopted to analyze the change of slope stability coefficient with rainfall duration, of which the calculation results were saved every 1 day for 14 days for both Rainfall I and Rainfall II to figure out the stability coefficient. The change of slope stability coefficient with rainfall duration under rainfall scheme I and II is shown in figure. The results show that: with the increase of rainfall duration, the stability of slope decreases continuously, and the impact of rainfall intensity on the safety coefficient is more significant, especially at the late stage of rainfall. What’s more, when the rainfall intensity increases from 180 to 260 mm/day, the slope safety coefficient under two different rainfall intensities decreases linearly with the rainfall duration from the early to the middle of the rainfall period. Under natural conditions, the slope safety coefficient is 1.184, which is in a stable state. as shown in Fig. 13. Under rainfall condition I, the slope safety coefficient decreases to 1.084, 1.029 and 1.012 on day 5, 10 and 14, respectively, while under rainfall condition II, the slope safety coefficient decreases to 1.078, 1.014 and 1.002 on day 5, 10 and 14, respectively. the change is 0.172 under rainfall condition I and 0.182 under rainfall condition II. The increase in rainfall intensity will lead to a decrease in the stability of the slope over time, and finally tends to 1 under rainfall condition II, which is likely to result in the destabilization of the whole slope.

**Figure 14:**
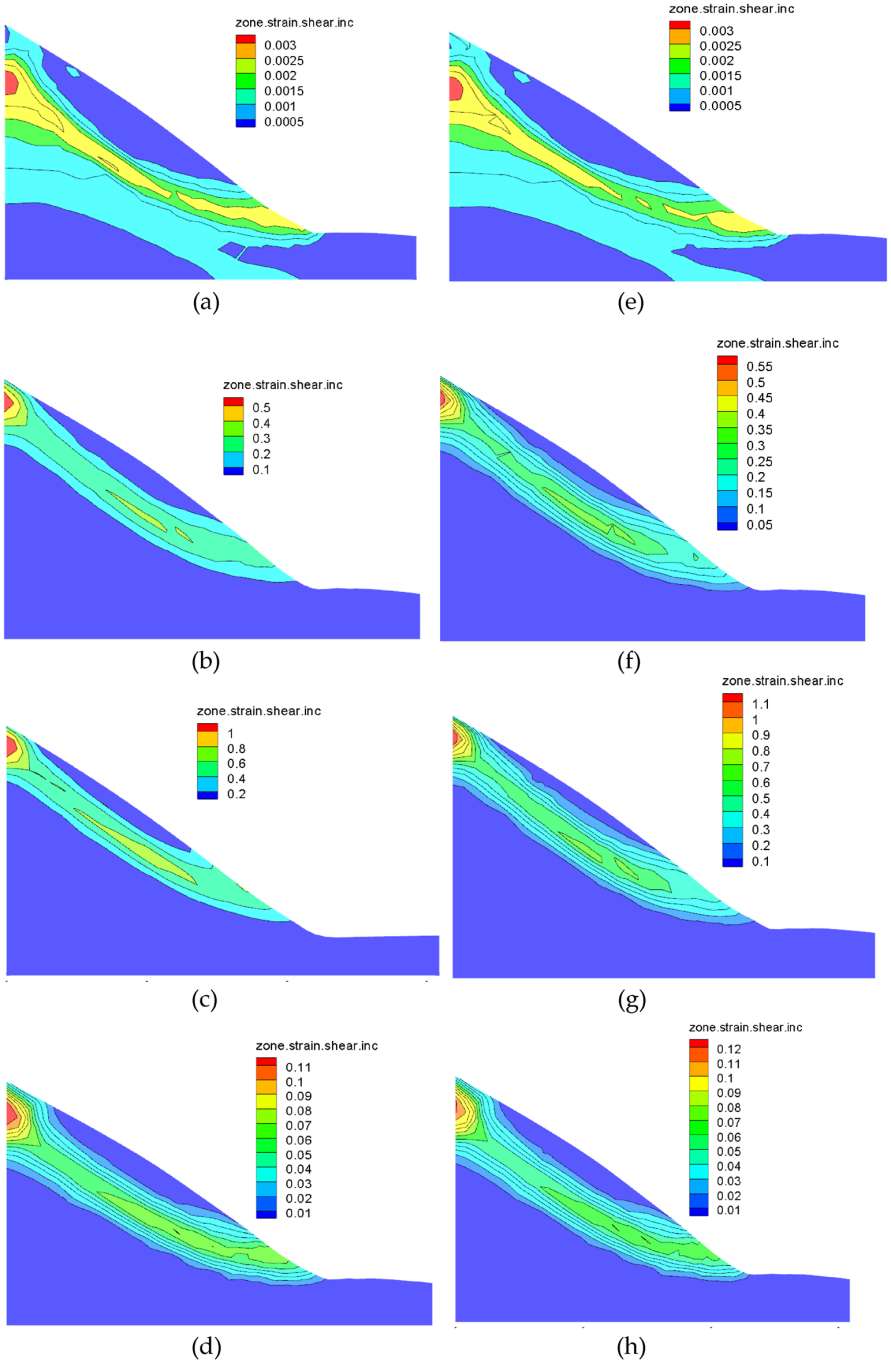
Shear strain diagram under different rainfall conditions: (**a**) rainfall I conditions on the first day; (**b**) rainfall I conditions on the 5th day; (**c**) rainfall I conditions on the 10th day; (**d**) rainfall I condition of the 14th day; (**e**) rainfall II conditions on the first day; (**f**) rainfall II conditions on the 5th day; (**g**) rainfall II conditions on the 10th day; (**h**) rainfall II conditions on the 14th day.

From Fig. 14a–d, it can be seen that the maximum shear strain value increases continuously as the accumulation of rainfall time increases. A large amount of rainwater infiltrates into the slope surface. The plastic zone of the slope develops continuously, and a large number of them are distributed on the surface, foot of the slope and slip zone of the slope.

Comparing with Figure 14a–h. with the increase of rainfall intensity, the maximum shear strain increases, and the distribution of shear strain increments is more dispersed than the sliding channel at the beginning and more coherent with the end of rainfall.

In summary, the maximum shear strain tends to increase continuously with the accumulation of rainfall and the increase of rainfall intensity. More specifically the higher the rainfall intensity, the faster the increase of the maximum shear strain value and the more concentrated the distribution of shear strain increments.

### Analysis of downslope displacement under rainfall conditions

**Figure 15:**
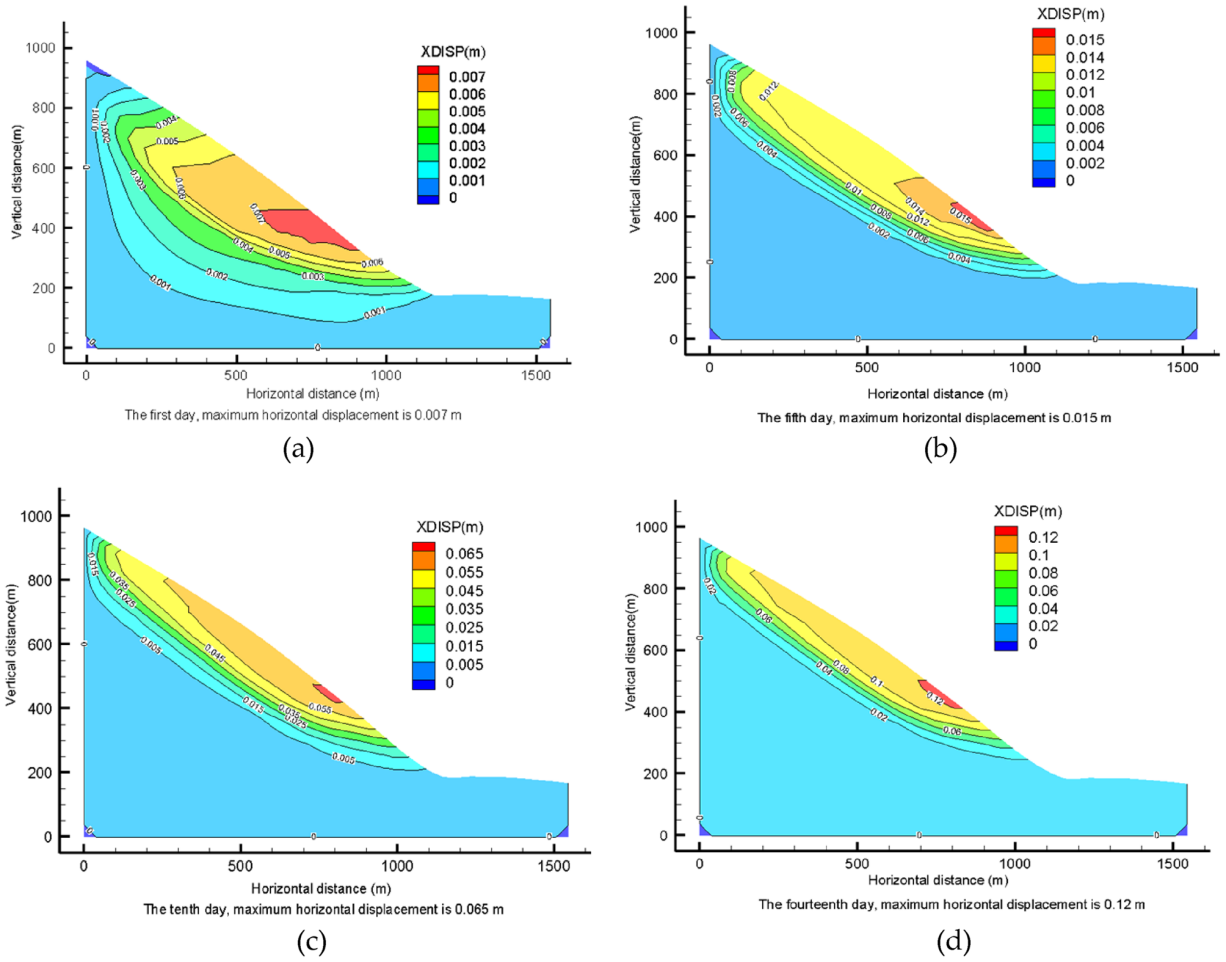
Horizontal displacement of lower slope under rainfall I conditions: (**a**) horizontal displacement of the slope on the 1st day of rainfall; (**b**) horizontal displacement of the slope on the 5th day of rainfall; (**c**) horizontal displacement of the slope on the 10th day of rainfall; (**d**) the horizontal displacement of the slope on the 14th day of rainfall.

On the first day of rainfall, the maximum horizontal displacement of the slope appears on the central surface of the slope. With the extension of rainfall time, the maximum horizontal displacement of the slope gradually shifts to the foot of the slope. On the 5th day of rainfall, the maximum horizontal displacement appears near basically follows the same pattern. After that, on the 10th and 14th day, the distribution of horizontal displacement is more or less the same, but the maximum horizontal displacement of the side slope gradually increases (Fig. 15a–d). Overall the horizontal displacement of the side slope mainly occurs in the upper part of the soil body, and increases gradually from the inside to the surface of that slope. At the beginning of the rainfall, the maximum horizontal displacement appears in the middle surface of the slope. Then, with the extension of rainfall time, the maximum horizontal displacement gradually increases. Finally, during the 14 days of rainfall, the horizontal displacement of the slope foot gradually increases, resulting in the gradual decrease in the slope stability.

### Rainfall condition II downslope body displacement analysis

**Figure 16:**
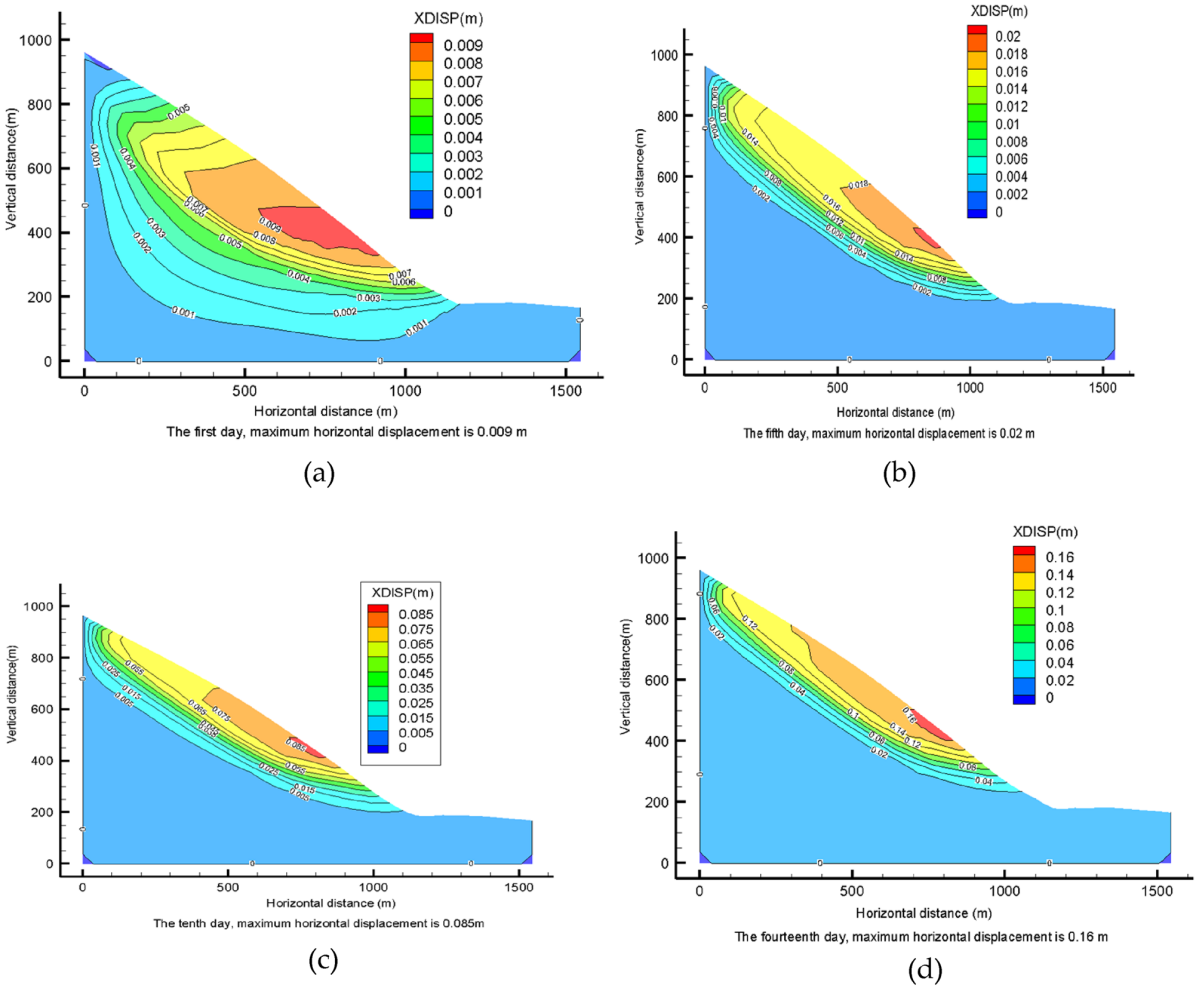
Horizontal displacement of lower slope under rainfall II conditions: (**a**) horizontal displacement of the slope on the 1st day of rainfall; (**b**) horizontal displacement of the slope on the 5th day of rainfall; (**c**) horizontal displacement of the slope on the 10th day of rainfall; (**d**) the horizontal displacement of the slope on the 14th day of rainfall.

On the first day of rainfall, the maximum horizontal displacement of the slope appears at the central surface of the slope. The rainfall value becomes larger, and the maximum horizontal displacement of the slope is also larger compared with the rainfall conditions. After that, the maximum horizontal displacement of the slope increases gradually on the 5th, 10th and 14th days of rainfall, but the distribution of horizontal displacement basically follows the same pattern (Fig. 16a–d). The maximum horizontal displacement is 0.16m on the 14th day.

### Coupling analysis of rainfall landslides

**Figure 17:**
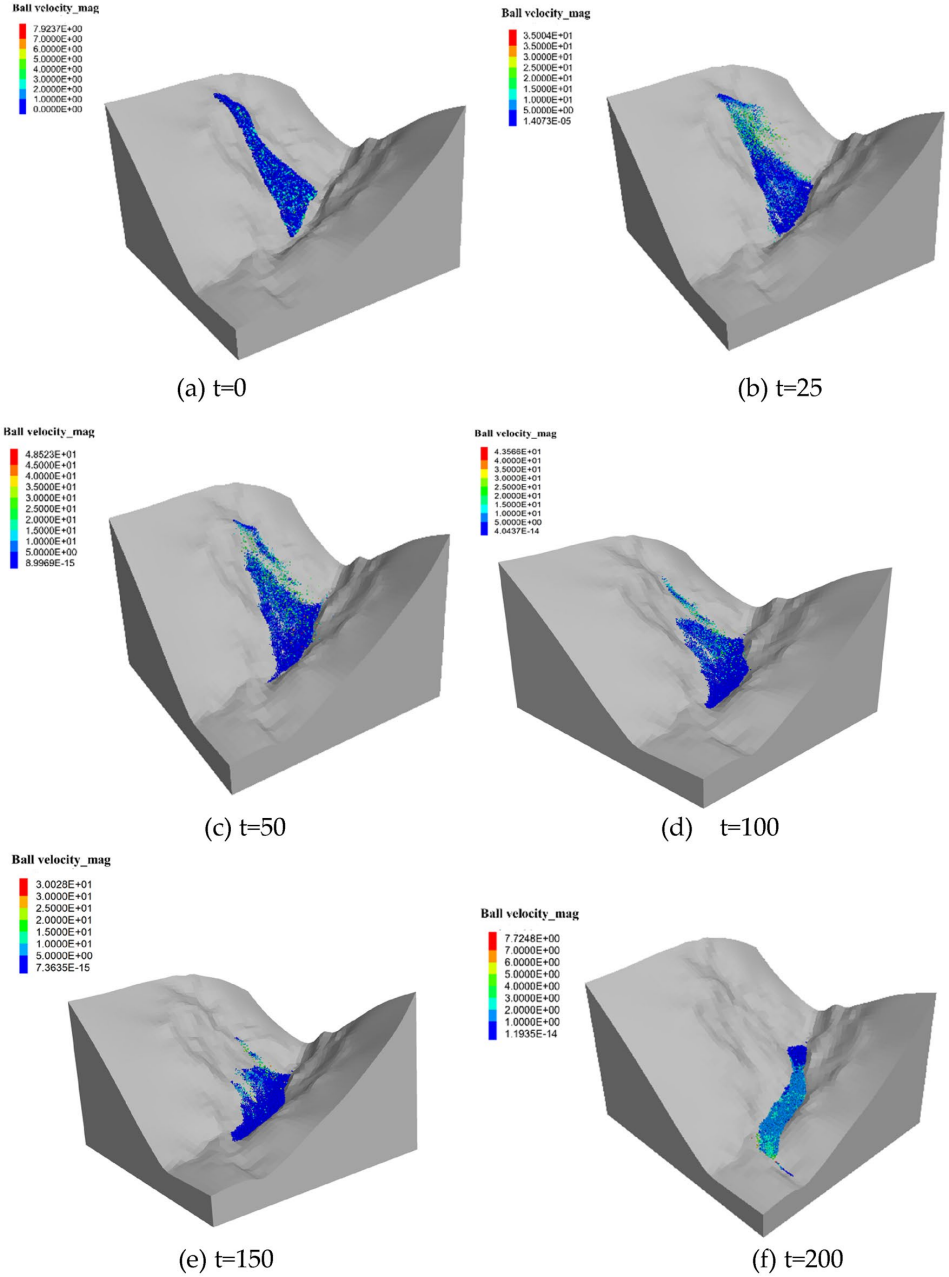
Dynamic process of the Ni changgou landslide (velocity nephogram): (**a**) t = 0 s; (**b**) t = 25 s; (**c**) t = 50 s; (**d**) t = 100 s; (**e**) t = 150 s; (**f**) t = 200 s.

With the purpose of doing the simulation on the landslide sliding process, the rainfall results are saved to the internal cell through boundary wall that is coupled to the continuous–discontinuous coupling model, and the rainfall loads are applied to the continuous and discontinuous models through equal effectiveness. It is that contact between the discrete particles and the cell grid is not close when the initial state gets generated leading to uneven coupling, thus the contact forces are not uniform, and the maximum displacement is generated in the vicinity of the vertical coupling intersection, the displacement no longer develops until the model gradually equilibrates and transmits uniform contact forces, and the calculation results of the rainfall landslide model are shown in Fig. 17.

Under the influence of continuous rainfall and gravity, the landslide moves downward rapidly. The whole process lasts about 200 s.

**Figure 18:**
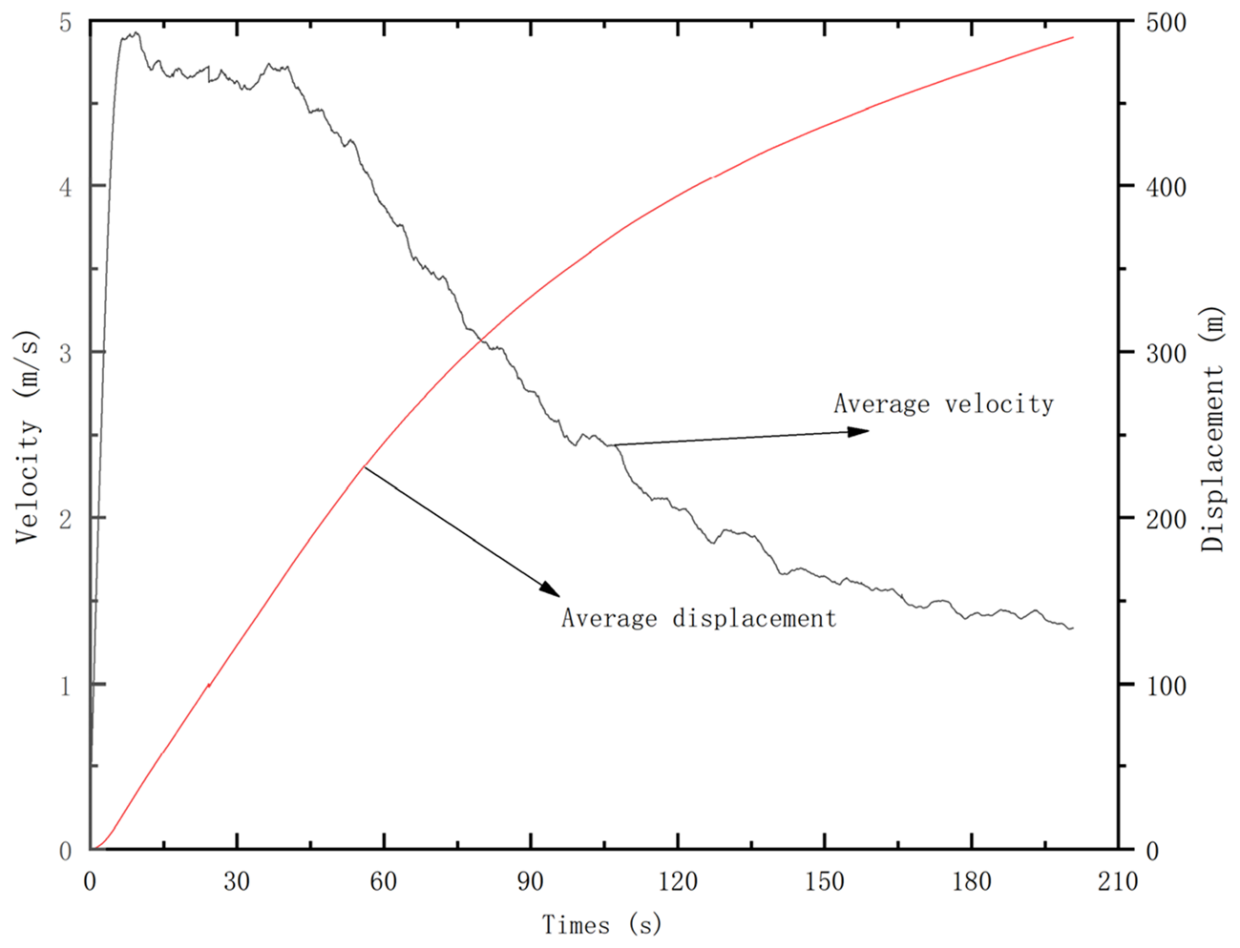
Average velocity and displacement evolution during different stages.

It can be divided into five stages: (1) At the initial moment, the slope is damaged along the sliding surface and show a trend of downward displacement (Fig. 17a). (2) 0–25 s, the head edge first separates from the slope surface and the sliding body moves rapidly to the lower ground level, and at the same time the landslide speed increases rapidly. The trailing edge part slides relatively slowly, with the maximum velocity exceeding 35 m/s, and the average velocity also increases rapidly to about 25 m/s. The remaining part of the sliding mass gathers and keeps moving downward (Fig. 17b). (3) During 25–50 s, the head edge portion separates from the slope and starts to accumulate downstream, and its velocity also gradually decelerates to 7–10 m/s. The back edge of the slider forms a distinct steep slope. The remaining sliding blocks continue to move downstream until they reach the channel and gradually accumulated (Fig. 17c). (4) During the propagation of the slide, the velocity gradually decreases due to the collision and friction between the particles. more and more sliding blocks get accumulated in the downstream channel (Fig. 17d, e). (5) After 200 s, most of the sliding mass stops moving and get accumulated at the foot of the slope, and only a small amount of sliding mass continues to move downstream. Finally, the sliding mass is deposited along the channel (Fig. 17f). The average velocity and displacement time courses of the soil at different stages are shown in Fig. 18, and the velocity variation in each stage is consistent with Fig. 17. Throughout the process, the landslide body velocity peaks in the second stage at 4.8 m/s, and the average jump distance of the landslide was close to 500 m.

### Motion process analysis

**Figure 19:**
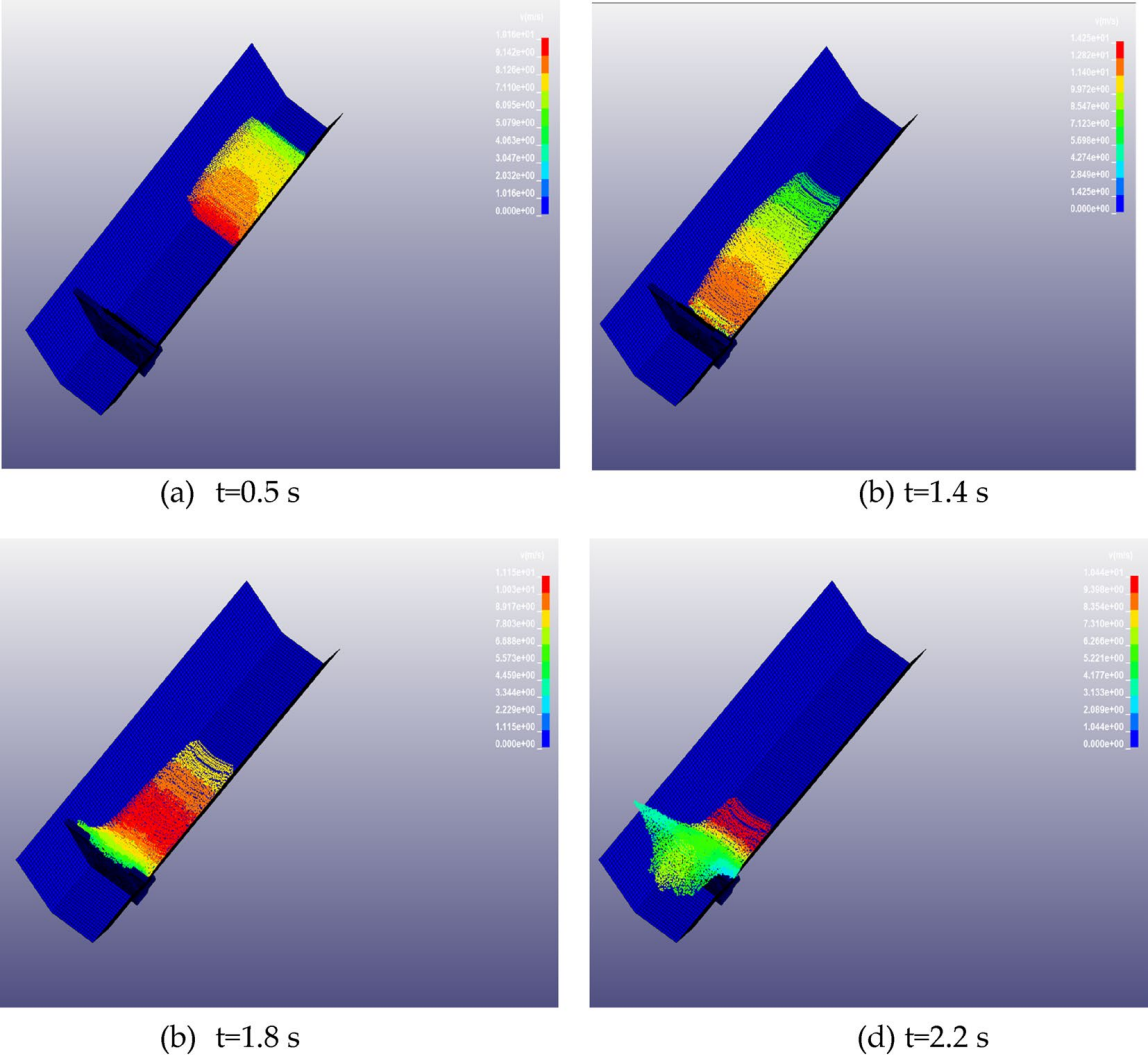
The whole process of debris flow on impact blocking structure.

To analyze the interaction process between the debris flow and the barrier dam, Fig. 19 presents the detailed process of debris flow impacting the barrier dam. When t = 0.5 s, the debris flow starts to move under the action of gravity, at this time the debris flow pattern has basically formed (Fig. 19a). When t = 1.4s, the debris flow reaches the bottom of the barrier structure, at this moment the mudslide began to impact the blocking structure and begins to climb higher (presented in Fig. 19b), the maximum velocity is 14.25 m/s. At t = 1.8s, most of the debris flow has stopped, but the debris flow still has an impact on the stopping structure (Fig. 19c). When t = 2.2 s, the debris flow partly overflows and partly flows back to siltation, and exerts a load effect on the stopping structure (Fig. 19d).

**Figure 20:**
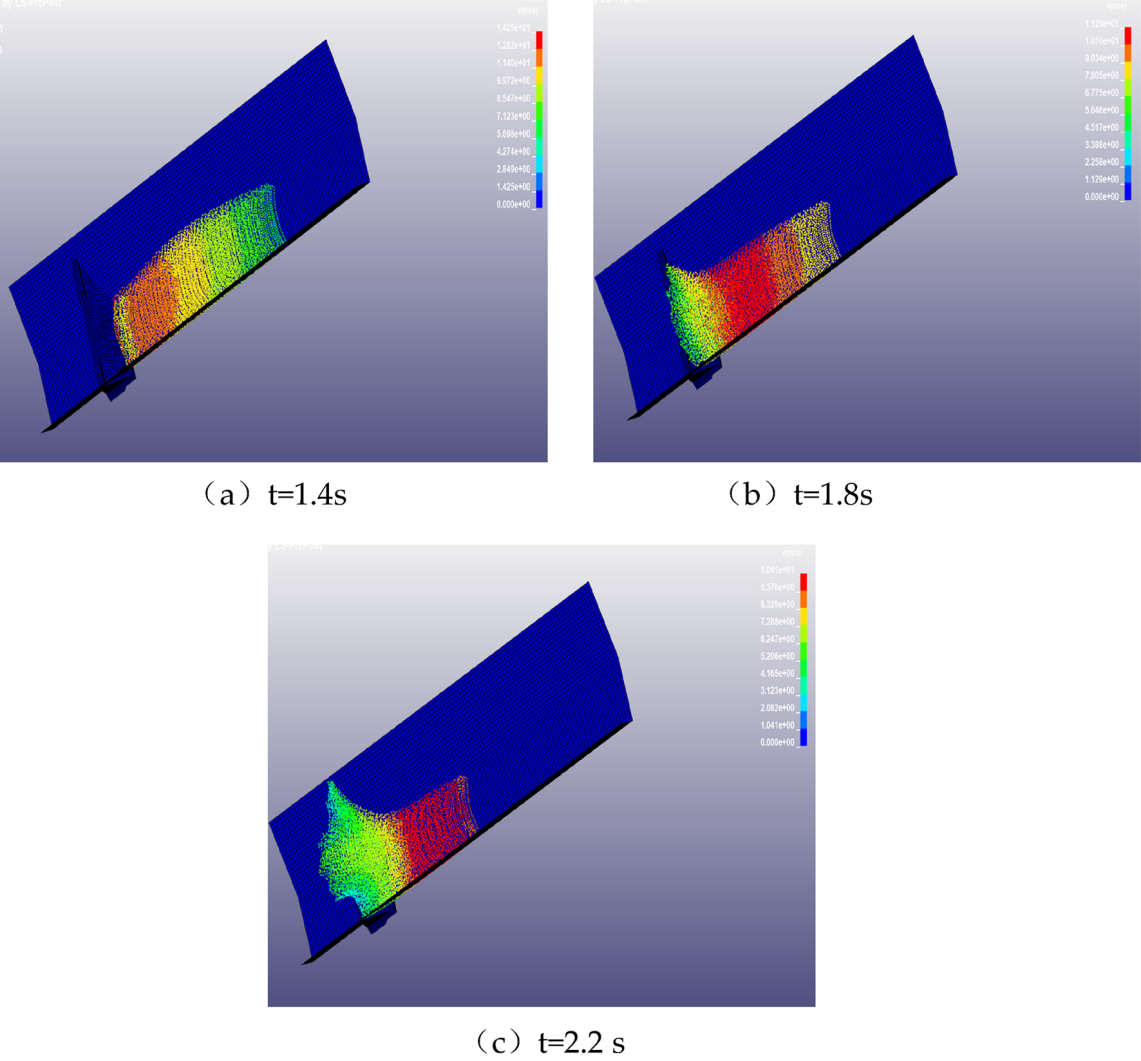
The process of climbing high and back silting of the blocking structure.

The process of climbing and desilting is shown in Fig. 20. At t = 1.4 s, the debris flow reaches the bottom of the barrier structure and starts to impact the debris flow barrier structure and starts climbing, the climbing height is 0.7 m (Fig. 20a); At t = 1.8 s, the debris flow continues to climb to a height of 3.7 m (see Fig. 20b) with local desilting, at this time the SPH fluid particle splash and diffusion phenomenon can be observed; At t = 2.2 s, the debris flow climbs to the highest with obvious desilting when it impacts the barrier structure, the climbing height is 3.7 m; finally, the debris flow climbs to the highest with obvious back-siltation and particle splash phenomenon when it impacts the barrier structure, and the climbing height reaches up to 5.7 m (Fig. 20c).

### Impact analysis

**Figure 21:**
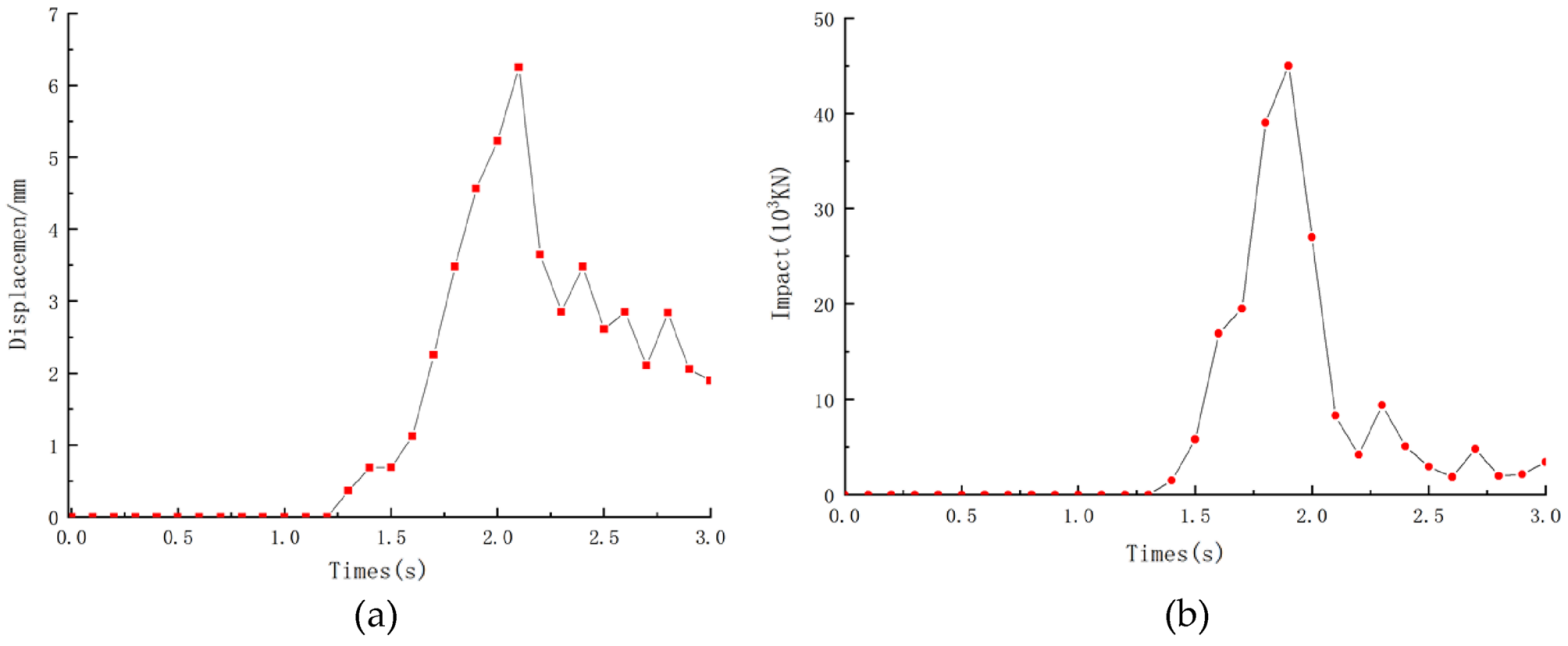
(**a**) Time course of displacement in the middle position of the top of the barrier structure; (**b**) time range of impact force of blocking structure.

**Figure 22:**
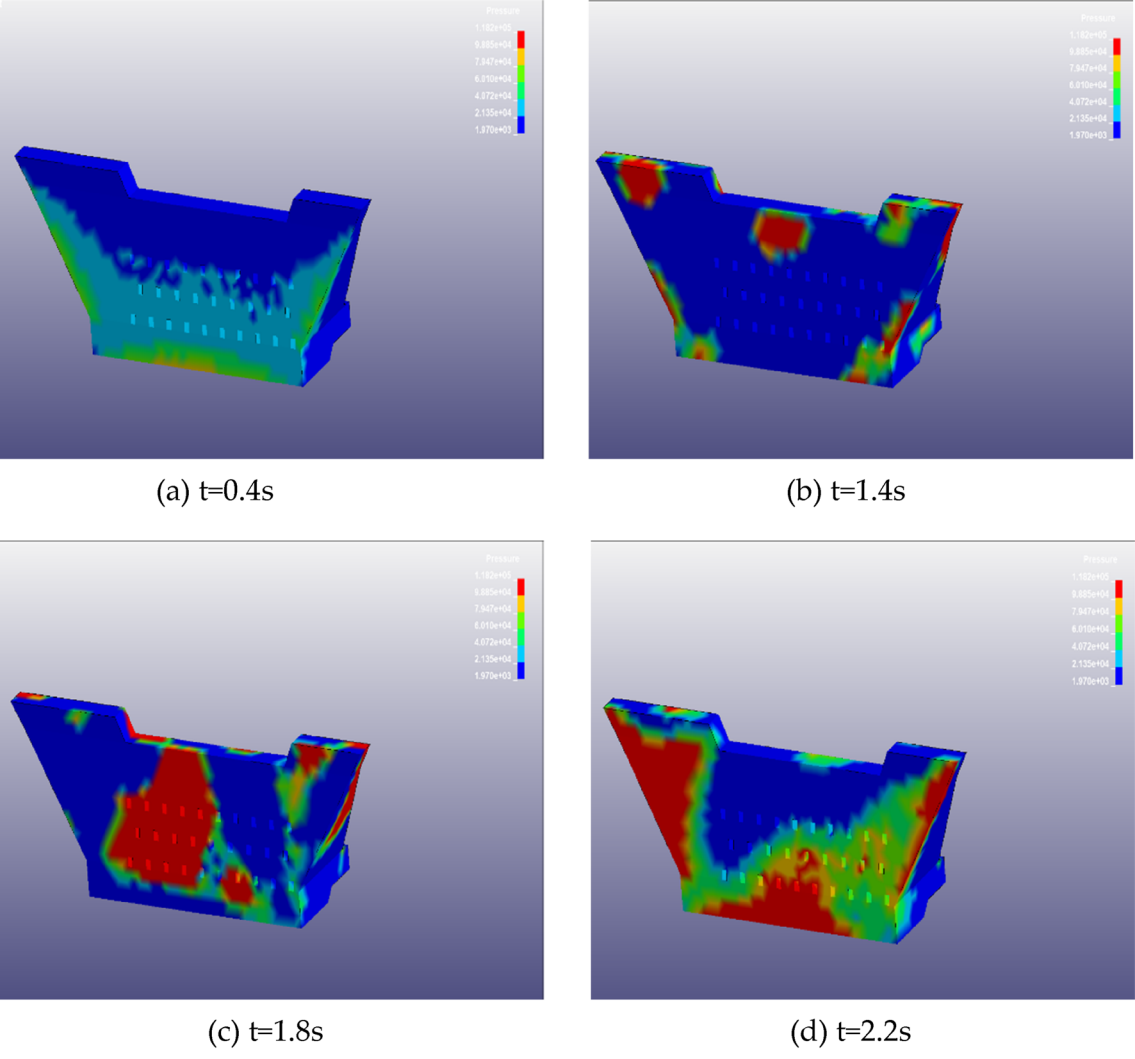
Blocking structure impact force distribution at different moments: (**a**) t = 0.4 s; (**b**) t = 1.4 s; (**c**) t = 1.8 s; (**d**) t = 2.2 s.

The displacement and time range of the middle of the blocking structure are monitored and shown in Fig. 21a. At about 2.2 s, the displacement of the middle of the blocking structure reaches a maximum value of 6.25 mm and rapidly decreases to 4.18 mm after reaching the sealed value, which is illustrated in Fig. 21a. And the subsequent impact force at different moments is shown in Fig. 21b. At t = 1.8 s, the peak impact force is 44,500 kN, and then rapidly decreases to 28,900 kN (see Fig. 21b), at this moment the impact force distribution of the blocking structure is shown in Fig. 22.

**Figure 23:**
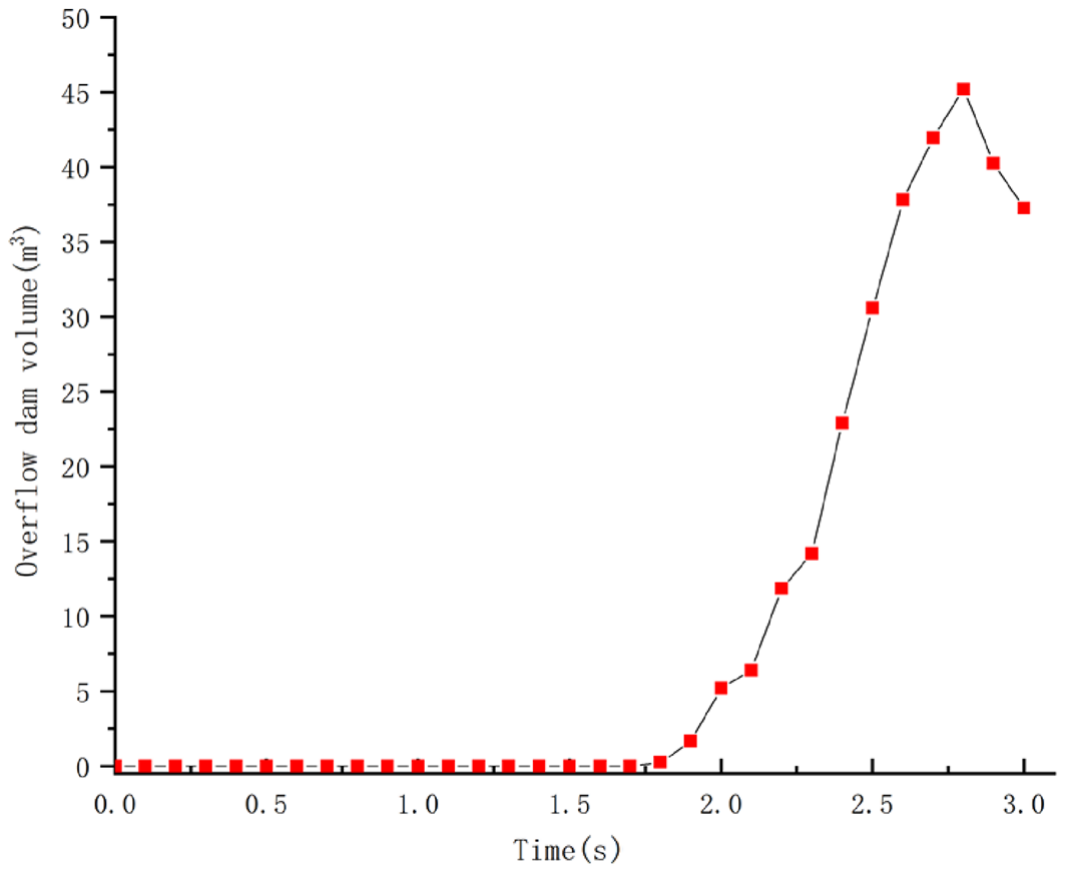
Variation of spill volume of blocking structure.

The debris flow slides along the trench, and a certain amount of debris flow will overflow in the process of hitting the dam, but it will conduct a certain back siltation state due to the dam's blocking effect, the change of volume is shown in Fig. 23. When t = 1.8 s, a certain amount of particles start to fly out, and at t = 2.8s, the overflow volume reaches the largest, with 46.73 m^3^, and then slowly flows back to siltation. It makes some reference significance for the dam's design of debris flow interception.

## Discussion

This contribution, using Ni changgou landslide as a focal point, solves into a further research of the landslide’s sliding mechanism through the utilization of numerical simulation. Additionally, it explores the influence of the landslide mudflow on the dam, employing a simplified terrain model. The findings are anticipated to provide significant insights for practical applications.

The research area under scrutiny has also the condition that breeds the occurrence of debris flow, and the downstream area has residents, roads, and farmland. Therefore, the design of the check dam becomes crucial. This study seeks to replicate the formation process of "dragon's head, dragon's body and dragon's tail" of the debris flow. The outcomes are expected to provide substantial guidance for the check dam design.

It is imperative to note that the research methodology outlined in this paper adopted a simplified terrain as the application scenario. However, in practical situations under other geological conditions, this study draws from the real context as a point of reference, conducting analyses to enhance the applicability and robustness of the proposed findings.

### Landslide analysis in other geological settings

**Figure 24:**
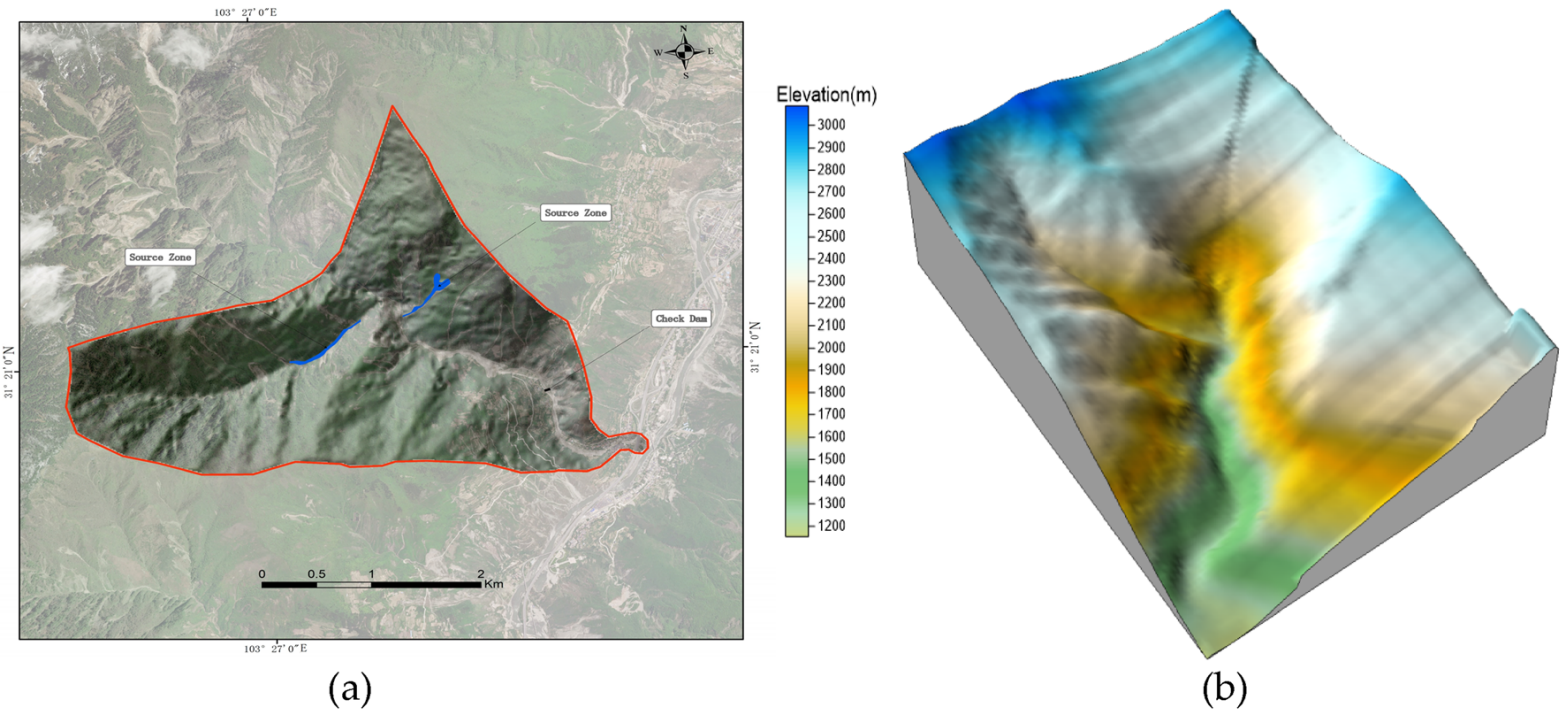
(**a**) Overview of the study area. (**b**) 3D topographic map of the Chutougou debris flow.

Chutougou is located in Wenchuan County, China, The Hotougou watershed is fan-shaped, and the bank slopes on both sides of the ditch are dominated by steep slope landforms (Fig. 24a), rainfall provides favorable conditions for the origin of debris flow.

After on-site investigation, it is shown that the hoe ditch treatment project is mainly based on the treatment idea of "mainly blocking and draining". As shown in Fig. 24b, 3D topographic map of the Chutougou is displayed.

### Chutougou debris flow simulation

**Figure 25:**
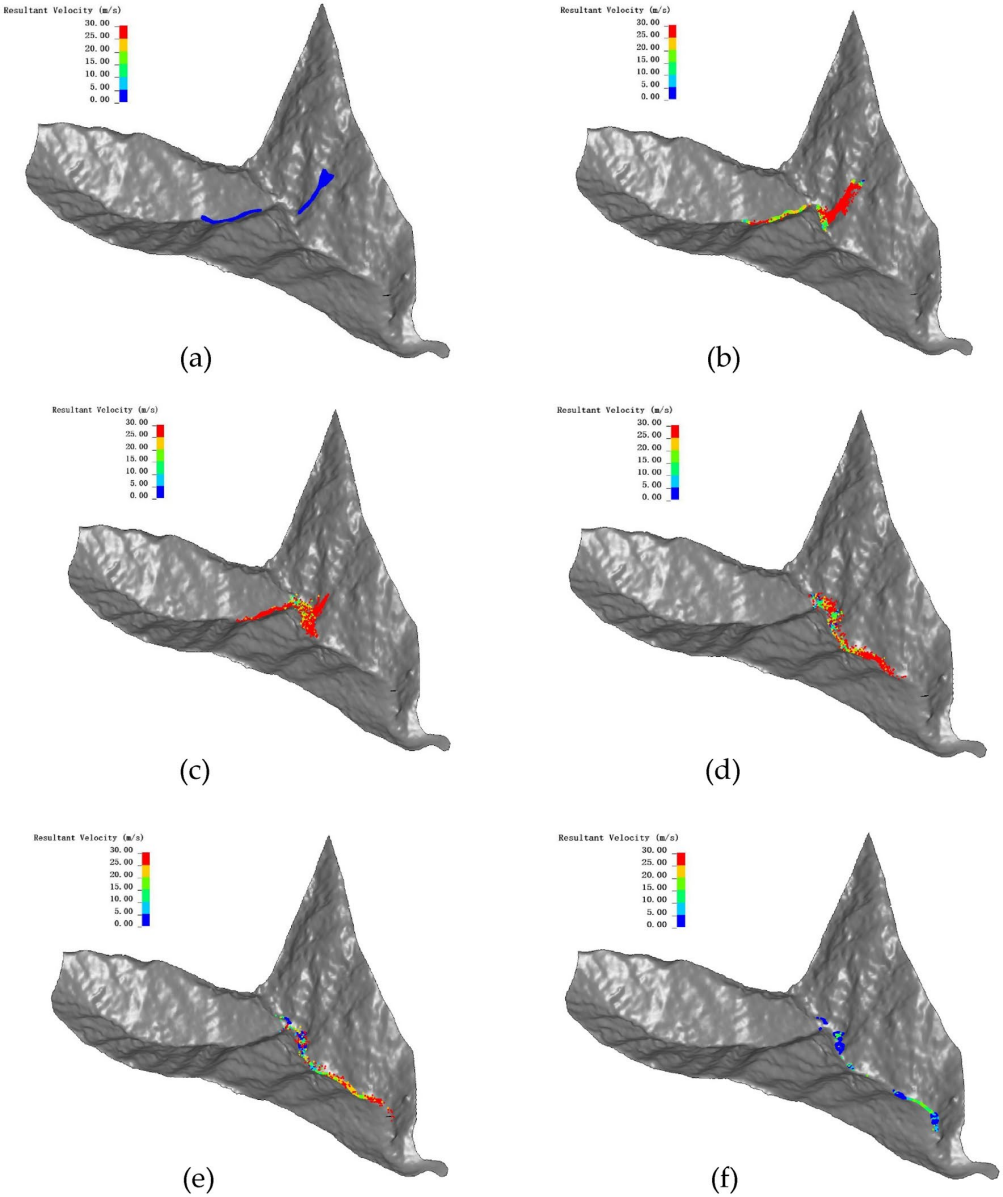
Velocity–time history of the simulated Chutougou debris flow.

Based on the SPH-FEM model applied in this paper, the SPH particle model is used to simulate the Chutougou debris flow, and the dam and topography are mainly simulated by the FEM model, according to the design parameters as shown in the following Table 3. The simulation speed is shown in the Fig. 25 below.

**Table 3:** Material models and parameters used in numerical simulation of Chutougou debris flow event.

Parameters	Value	Value
Topographic surface	Young’s modulus (GPa)	230
	Poisson ratio	0.2
	Density (kg/m^3^)	2800
Debris particles	Cohesion (kPa)	15
	Poisson ratio	0.3
	Density (kg/m^3^)	1900
	Sliding friction coefficient	0.25
	Initial porosity	0.5
Check dam	Density (kg/m^3^)	2500
	Young’s modulus (GPa)	25
	Poisson ratio	0.3
	Shear modulus (MPa)	0

The initial state is shown in Fig. 25a. The Chutougou debris flow has a relatively high velocity at the beginning As shown in Fig. 25b, which may be related to the large number of landslides and the steep terrain As shown in Fig. 25c, d after the debris flow slides to the main ditch, the velocity increases significantly, and quickly rushes downstream, all the flowing materials then slow down and accumulate downstream, and the debris flow increases at the bend, as shown in Fig. 25e, the debris flow begins to contact the check dam, the flow velocity decreases to the position where the dam body is located, due to continuous accumulation, and finally occluded, with the arrival of the subsequent debris flow, part of the debris flow material is intercepted in the retaining dam, and the remaining material flows downstream through the retaining dam. as shown in Fig. 25f

**Figure 26:**
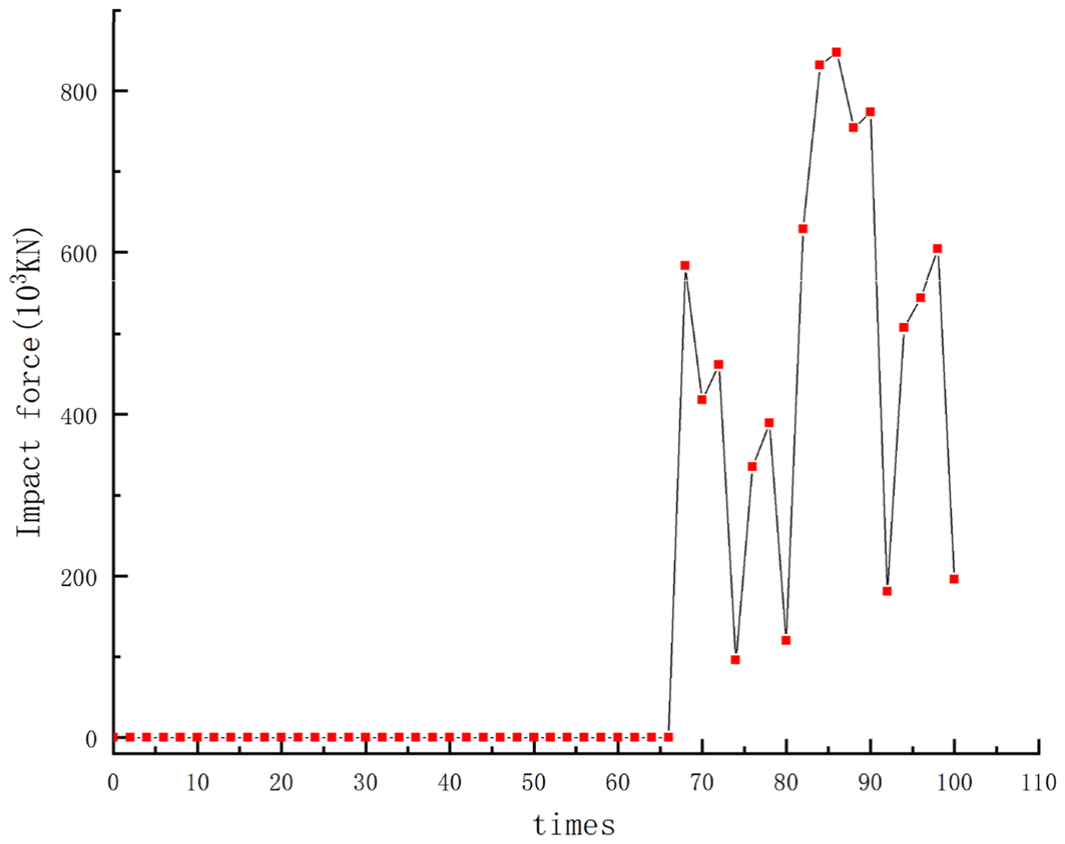
Impact force evolutions of the comb dams.

The evolution of the impact force is shown in Fig. 26, shows the time history curve of the Impact force when the debris flow made contact with the structure. The impact force of the debris flow on the dam is 823 × 10^3^ kN, and the debris flow changes from high-speed particles to low- speed particles through the dam blocking effect, has a significant effect on the energy dissipation of the debris flow.

**Figure 27:**
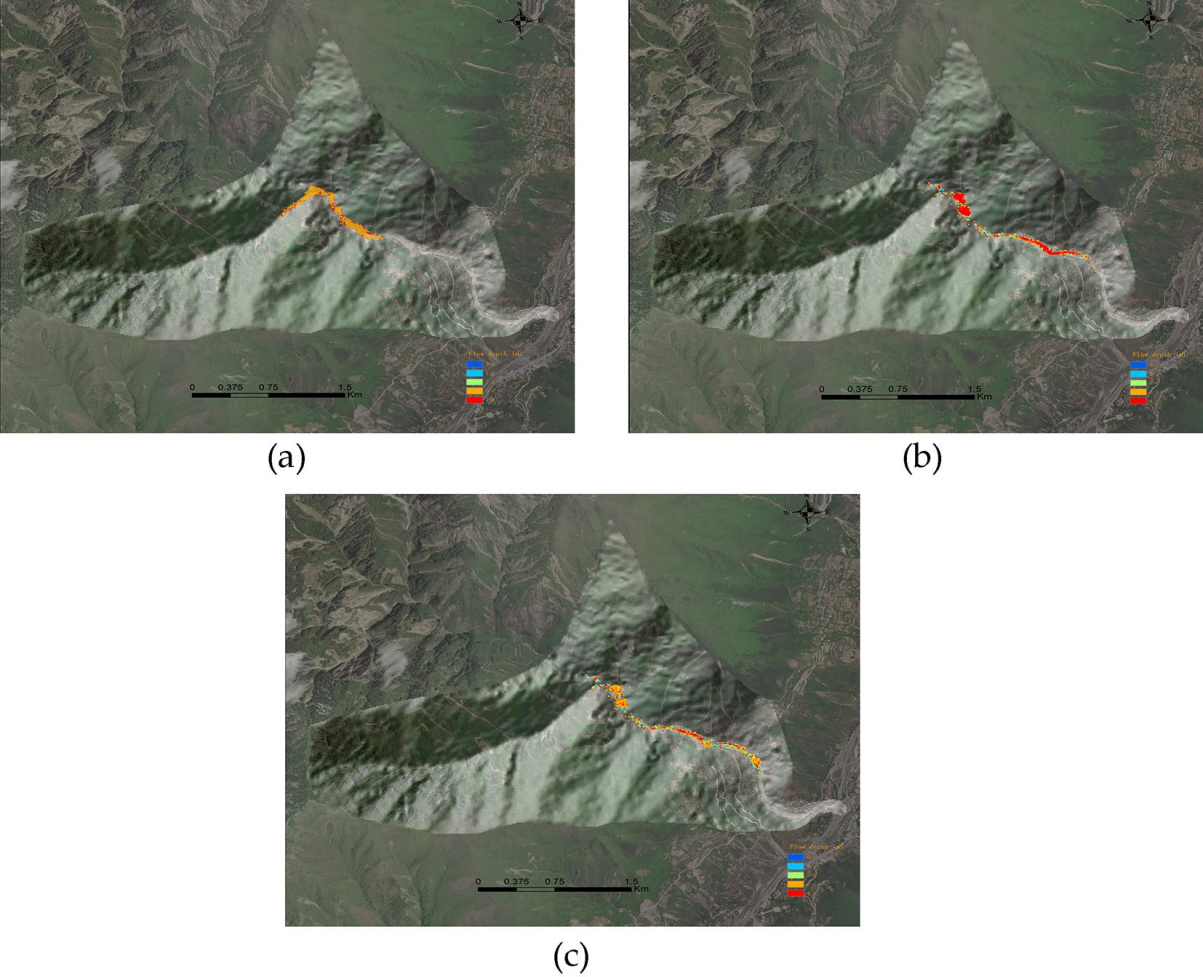
Simulated flow height of the Chutougou debris flow.

The simulated flow depth of the debris flow is shown in Fig. 27. After a mudslide, solids accumulate in the river channel and rush downstream. In about 20 s (Fig. 27a), a fast-moving debris flow of branches begins to flow into the main channel. and quickly transported along the main channel. As can be seen from Fig. 27b, the debris flow reached the location of the hoe ditch dam in about 60 s, and the debris rushed out of the dam along the channel as the upstream debris flow continued to flow forward along the channel. Depths of up to 5 m were reached in several places, as shown in Fig. 27c. After moving for about 100 s, the debris flow stops and accumulates in the middle and lower reaches. The morphology and thickness of the final sediment are shown. The thickness of the sediment at the outlet of Hongchungou is 0–5 m. In this study, considering the characteristics of the local region, the classification standard for debris flow intensity that considers both the siltation damage and impact damage is generated (Table 4) based on the suggestions in Ouyang et al.^44^ the debris flow-affected areas can be divided into three intensity levels (low, medium, and high).

**Table 4:** Classification standard for debris flow intensity.

Debris flow intensity	Maximum flow height h (m)
High	H ≥ 4.0
Medium	1.5 ≤ h < 4.0
Low	0 ≤ h < 4.0

This paper reproduces the movement process of debris flow by taking the hoe debris flow in other regions as a practical example, and further analyzes the spatial information carried by the example in the risk assessment, and obtains the hazard intensity of the debris flow, which can provide a better basis for disaster analysis and disaster reduction design.

## Conclusions

This paper takes the Ni changgou landslide mound as an example, establishing a three-dimensional model that accounts for rainfall. A comprehensive series of numerical studies is conducted, employing the unsaturated seepage analysis method to analyze the variation law of pore water pressure under the action of rainfall infiltration. Furthermore, the investigation extends to address the impact force problem, dynamic response problem related to debris flow, and its blocking structure has been conducted, the model of the debris flow in hoe ditch was verified, and the risk assessment was carried out and the following conclusions are drawn:

1. Ni changgou landslide, located in a shallow surface stratum and recognized as a landslide-prone zone, is triggered by continuous heavy rainfall leading to slope damage and subsequent landslides. The descending slope slides, following the landslide, propels into the river, culminating in a mudslide that inflicts considerable damage to the surrounding environment.
2. Long-term continuous rainfall result in a progressively increasing maximum horizontal displacement of the slope, corelating with both the extension of rainfall time and the increase of rainfall intensity. The maximum horizontal displacement of the slope appears in the middle of the slope at the onset of rainfall. At the end of rainfall, the maximum horizontal displacement of the slope under rainfall condition II is 0.16 m.
3. Analysis of saturated-unsaturated rainfall infiltration involves a continuous model, establishing a continuous-discontinuous coupling model through a boundary wall coupling method. Additionally, the seepage field is equivalently applied to the coupling model equivalently, facilitating landslide coupling analysis under the influence of rainfall. The simulation results depict the entire process of Ni changgou landslide lasts about 200 s. It can be divided into five sub-stages: early accelerated deformation stage, slider trailing edge disintegration stage, jumping stage along the main slide surface, deceleration movement stage and final deposition stage. The average velocity of the landslide can reach 4.85 m/s, with an average displacement of approximately 500 m. After the movement ceases, the landslide gets accumulated along the river basin hillside.
4. The three-dimensional simulation of the Chutougou debris flow in hoe ditch was carried out through the coupled model, yielding the maximum depth of the debris flow accumulation. The dam exhibits a certain blocking effect on the debris flow, which could provide theoretical support and technical basis for the prediction and evaluation of debris flow disasters.

However, although the movement process of Ni changgou and Chutougou debris flow is simulated by SPH-FEM coupling method, the coupling calculation model still has shortcomings, and the influence of pore water pressure (rainfall) is ignored in the process of movement, and it will be further improved in the future.

## Data Availability

The datasets used or analysed during the current study available from the corresponding author on reasonable request.
